# Hyperdominant Trees Reveal Savanna Vulnerability Under Climate Change

**DOI:** 10.1111/gcb.70859

**Published:** 2026-04-16

**Authors:** Facundo Alvarez, Ben Hur Marimon‐Junior, Beatriz Schwantes Marimon, Wesley Jonatar Alves da Cruz, Norberto Gomes Ribeiro Júnior, Raiane Gonçalves Béu, Polyanna da Conceição Bispo, Aldair de Souza Medeiros, Glécio Machado Siqueira, Marcelo Leandro Bueno, Fabiana de Gois Aquino, Frederico Augusto Guimarães Guilherme, José Roberto Rodrigues Pinto, Henrique Augusto Mews, Bruno Machado Teles Walter, Sabrina do Couto de Miranda, Ricardo Flores Haidar, Eddie Lenza de Oliveira, Renata Dias Françoso Brandão, Eraldo Aparecido Trondoli Matricardi, Cássia Beatriz Rodrigues Munhoz, Edson de Souza Lima, Maria Antônia Carniello, Mercedes Maria da Cunha Bustamante, Paulo Sérgio Morandi, Edmar Almeida de Oliveira, Zenésio Finger, Eder Carvalho das Neves, Fernando Elias, Immaculada Oliveras Menor, Simone Matias de Almeida Reis, Oliver Phillips, Ted R. Feldpausch

**Affiliations:** ^1^ Universidade do Estado de Mato Grosso, Campus de Nova Xavantina Mato Grosso Brazil; ^2^ Departamento de Geociências Universidade Federal do Maranhão São Luís MA Brazil; ^3^ AMAP – botAnique et Modélisation de l'Architecture des Plantes et des Végétations Université de Montpellier, CIRAD, CNRS, INRAE, IRD Montpellier France; ^4^ Department of Geography, School of Environment, Education and Development University of Manchester Manchester UK; ^5^ Laboratório de Macroecologia e Evolução Universidade Estadual de Mato Grosso do Sul Campo Grande Mato Grosso do Sul Brazil; ^6^ Embrapa Cerrados Brasília DF Brazil; ^7^ Instituto de Biociências, Departamento de Biodiversidade Universidade Federal de Jataí Jataí Goiás Brazil; ^8^ Departamento de Engenharia Florestal Universidade de Brasília Brasília DF Brazil; ^9^ Instituto de Ciências Exatas e Naturais Universidade Federal de Rondonópolis Rondonópolis Mato Grosso Brazil; ^10^ Embrapa Recursos Genéticos e Biotecnologia Herbário CEN Brasília DF Brazil; ^11^ Unidade Universitária de Palmeiras de Goiás Universidade Estadual de Goiás Goiás Brazil; ^12^ Curso de Engenharia Ambiental Universidade Federal do Tocantins Palmas Tocantins Brazil; ^13^ Universidade Federal de Lavras Lavras Minas Gerais Brazil; ^14^ Universidade de Brasília Departamento de Botânica, Instituto de Ciências Biológicas Brasília DF Brazil; ^15^ Universidade do Estado de Mato Grosso Mato Grosso Brazil; ^16^ Departamento de Ecologia Universidade de Brasília Brasília DF Brazil; ^17^ Faculdade de Engenharia Florestal Universidade Federal de Mato Grosso Mato Grosso Brazil; ^18^ Embrapa Amazônia Oriental Belém Pará Brazil; ^19^ Centro de Ciências Biológicas e da Natureza Universidade Federal do Acre Rio Branco AC Brazil; ^20^ School of Geography University of Leeds Leeds UK; ^21^ Geography, Faculty of Environment, Science and Economy University of Exeter Exeter UK

**Keywords:** Cerrado, conservação, fogo, árvores hiperdominantes, savana, modelos de distribuição de espécies, Cerrado, conservation, fire, hyperdominant trees, savanna, species distribution models

## Abstract

The Cerrado biome, spanning ~2 million km^2^, is one of the most extensive and biodiverse tropical savannas, yet it is paradoxically dominated by only 30 hyperdominant tree species (~2% of all species, > 50% of all stems). However, their vulnerability to climate change and the effectiveness of current conservation efforts remain uncertain. By combining (i) species distribution models calibrated with edaphic‐climatic predictors and occurrence data for hyperdominant Cerrado trees with (ii) functional‐trait analyses related to ecological strategies (leaf economics, bark investment, ecophysiology, drought tolerance, regeneration, and dispersal), we identified the biomass production and reproductive traits that best predict species persistence under high‐emission scenarios (RCP8.5). Currently, only 427,980 km^2^ (~17.4%) of the modeled potential environmental suitability falls within protected areas; under future climate scenarios, this protected suitable area is projected to decline by ~45.9% to 231,377 km^2^ (~18.1%). This loss, resulting from land conversion with the expansion of agricultural frontiers and fire events, highlights a mismatch in current regional conservation priorities, overlooking the needs of the Cerrado and its associated biodiversity. Given ongoing land‐use change and that 58% of remaining native vegetation occurs on private lands, this mismatch highlights the urgent need to align conservation and agricultural policy. Functional trait analyses revealed a clear gradient from acquisitive “fast” to conservative “slow” strategies, reflecting trade‐offs in water‐use efficiency and biomass allocation, with leaf structural and reproductive traits best predicting species resilience to climate change. Our results indicate that a small subset of hyperdominant species not only forms current community structure but also signals the biome's resilience/vulnerability to climate change. Conservation planning should prioritize identified climate refuges and be based on functional traits to buffer the loss of functional and structural integrity in one of the world's richest and most threatened savanna ecosystems.

## Graphical abstract

The Brazilian Cerrado's forest structure relies disproportionately on just 30 hyperdominant tree species, which represent 50% of all individuals despite accounting for only 2% of species richness. This study evaluates their vulnerability under climate change scenarios for 2100. Our findings reveal that these vital species are highly threatened, with a projected 45.9% reduction in suitable habitat due to rising temperatures and increased aridity. Furthermore, we identify a critical conservation gap: less than 18% of high‐suitability habitats are currently within protected areas. By integrating leaf and reproductive functional traits, we demonstrate that specific ecological strategies are key predictors of climate resilience. These results underscore that protecting common, hyperdominant species is a fundamental yet overlooked priority for maintaining ecosystem stability and biome‐scale persistence in the face of global warming.

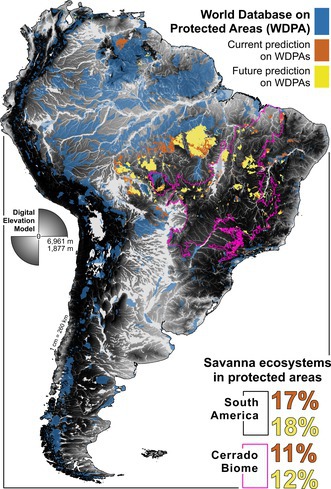

## RESUMO

O bioma Cerrado, abrangendo cerca de 2 milhões de km^2^, é uma das savanas tropicais mais extensas e biodiversas, mas é paradoxalmente dominado por apenas 30 espécies de árvores hiperdominantes (~2% de todas as espécies, > 50% de todos os troncos). No entanto, a vulnerabilidade dessas espécies às mudanças climáticas e a eficácia dos esforços de conservação atuais permanecem incertas. Ao combinar (i) modelos de distribuição de espécies calibrados com preditores edafoclimáticos e dados de ocorrência de árvores hiperdominantes do Cerrado com (ii) análises de traços funcionais relacionados a estratégias ecológicas (economia foliar, investimento em casca, ecofisiologia, tolerância à seca, regeneração e dispersão), identificamos os traços de produção de biomassa e reprodutivos que melhor preveem a persistência das espécies sob cenários de alta emissão (RCP8.5). Atualmente, apenas 427.980 km^2^ (~17,4%) da adequação ambiental potencial modelada estão dentro de áreas protegidas; sob cenários climáticos futuros, projeta‐se que esta área adequada protegida decline em ~45,9%, para 231.377 km^2^ (~18,1%). Essa perda, resultante da conversão de terras com a expansão das fronteiras agrícolas e eventos de fogo, destaca uma incompatibilidade nas prioridades regionais de conservação atuais, negligenciando as necessidades do Cerrado e sua biodiversidade associada. Dado que a mudança no uso da terra é contínua e que 58% da vegetação nativa remanescente ocorre em terras privadas, esse desajuste ressalta a necessidade urgente de alinhar as políticas de conservação e agrícola. As análises de traços funcionais revelaram um gradiente claro de estratégias “rápidas” (aquisitivas) a “lentas” (conservativas), refletindo compensações (trade‐offs) na eficiência do uso da água e na alocação de biomassa, com traços estruturais foliares e reprodutivos prevendo melhor a resiliência das espécies às mudanças climáticas. Nossos resultados indicam que um pequeno subconjunto de espécies hiperdominantes não apenas forma a estrutura atual da comunidade, mas também sinaliza a resiliência/vulnerabilidade do bioma às mudanças climáticas. O planejamento da conservação deve priorizar os refúgios climáticos identificados e basear‐se em traços funcionais para amortecer a perda da integridade funcional e estrutural em um dos ecossistemas de savana mais ricos e ameaçados do mundo.

## 1 Introduction

The Cerrado biome is a megadiverse phytogeographic domain. Covering approximately 2 million km^2^, it is among the most extensive and biodiverse savannas in the world, constitutes an important Neotropical biodiversity hotspot, and is one of the largest centers of endemism in Brazil (Myers et al. 2000; Strassburg et al. 2017; Alvarez et al. 2025). Despite its importance, the biome has lost 140,000 km^2^ of native vegetation in three decades and retains only about 58% of its original cover, so much of its remaining habitat is outside of formal protected areas (Alencar et al. 2020; Colman et al. 2022; Vieira et al. 2022). Understanding both how Cerrado biodiversity has been shaped by past biogeographical processes and how it will respond to future climate change requires identifying the species that are most critical to the biome's long‐term persistence. To inform conservation planning under heightened anthropogenic pressure and climate change, species distribution models (SDMs) offer a framework that integrates species' historical biogeography dynamics with future range shifts (Arroyo‐Rodríguez et al. 2020; Diniz‐Filho et al. 2020).

Quaternary climate changes strongly affected Cerrado biodiversity. The extraordinary diversity of the Cerrado reflects long‐term interactions among fire regimes, edaphic heterogeneity, and Quaternary climatic dynamics: Pleistocene refugia and local adaptation have contributed to high endemism (currently over 34%) and the evolution of distinctive functional strategies (Bueno et al. 2018; Durigan 2020). Cerrado tree species also exhibit the abundance–occupancy relationship: rare species are locally scarce and geographically restricted, while dominant species are widely distributed (Alvarez et al. 2025). Likewise, patterns of hyperdominance and the center–periphery hypothesis are observed in the Cerrado, with decreases in both diversity and abundance away from distribution centers (Pfeilsticker et al. 2021; Alvarez et al. 2025). In the Cerrado biome, only 30 hyperdominant tree species account for 50% of all trees. In comparison, the Amazon hosts 227 hyperdominant tree species (ter Steege et al. 2013). The small set of Cerrado hyperdominant tree (CHT) species in the Cerrado may constitute a potential vulnerability, increasing the risk that the biome loses its defining ecological characteristics, as these species are key architects of biomes and landscapes (ter Steege et al. 2013; Alvarez et al. 2025). Conversely, if they prove resilient, they may buffer community‐level losses.

Currently, the Cerrado faces both intense anthropogenic threats and significant knowledge gaps, including taxonomic (Prestonian‐Linnean), geographic (Hutchinsonian‐Wallacean), and functional (Raunkiærian) deficiencies, with ~1605 species still pending formal description (Alvarez et al. 2025). Furthermore, since 1985, the Cerrado biome has lost ~24 billion trees (Alvarez et al. 2025), and human actions have transformed it more rapidly than the Amazon, with an annual loss of vegetation 2.4 times greater than that of the Amazon in 2023 (MapBiomas 2024). Projections indicate that by 2030, most of the remaining Cerrado vegetation could persist almost exclusively within protected areas; currently, only 8.4% of native vegetation is under protection (Françoso et al. 2020; Vieira et al. 2022; Alvarez et al. 2025). However, conservation planning in the Cerrado is often reactive rather than strategic, with protected areas established opportunistically in response to immediate threats rather than being designed according to long‐term ecological priorities (Strassburg et al. 2017; Diniz‐Filho et al. 2020; Colman et al. 2022).

CHT resilience under rapid climate change remains uncertain. Future climates are expected to impose stronger drought stress and higher temperatures across the Cerrado (Hofmann et al. 2023), along with increased fire‐prone conditions driven by anthropogenic warming (Durigan 2020; Li et al. 2021). The extent to which these environmental shifts will challenge species' physiological limits and affect biome‐level resilience remains unclear, as the persistence or loss of a small set of hyperdominant species could substantially alter Cerrado structure and function (Strassburg et al. 2017; Alvarez et al. 2025). Therefore, the ecological strategies that arise from the interaction between functional traits and environmental factors act as fundamental links between biological processes and community dynamics (Souza et al. 2022). Functional traits differentially influence the survival, growth, and reproduction of species, and act as indicators for understanding macroecological responses to climate and land‐use change (Cruz et al. 2025). Understanding these traits improves our ability to anticipate vegetation dynamics in the face of rapid climate and environmental changes (Souza et al. 2022; Ribeiro‐Júnior et al. 2023). To assess this, we evaluated whether CHT constitute reliable indicators of biome‐scale resilience. We combined SDMs, future climate projections, protected area assessments, and functional‐trait analyses to (1) model current climatic suitability using SDMs, (2) project these models under future climate scenarios, (3) overlay suitability maps with protected area boundaries to assess current and future representation, and (4) relate projected suitability changes to species functional traits to infer resilience.

## 2 Material and Methods

### 2.1 Study Area

The Cerrado is the savanna that contributes most significantly to global biodiversity (Myers et al. 2000; Overbeck et al. 2022). The various vegetation types of the Cerrado predominantly occur on nutrient‐poor soils (Ribeiro and Walter 2008). According to the Brazilian Soil Classification System, these are mainly Oxisoils, Latosols and Quartzarenic Neosols, corresponding to Ferralsols and Arenosols in the World Reference Base (IUSS 2015). In contrast, fertile limestone‐derived soils in the region support seasonal deciduous forests rather than savannas (Bueno et al. 2018; Elias et al. 2019; Marimon‐Junior et al. 2019). The core area of the Cerrado biome is largely associated with the Central Plateau region (6°–17° S, 44°–53° W), where the climate is classified as Köppen Aw (tropical seasonal), with a rainy summer (October to April) and a dry winter (May to September). Regardless of tree physiognomy, most species are perennial and shade‐intolerant, having co‐evolved with fire (Silvério et al. 2013; Durigan 2020). Evidence suggests that the Cerrado emerged through recent and recurrent adaptations to fire, rather than through the arrival of fire‐preadapted lineages (Durigan 2020; Li et al. 2021), and that strong edaphoclimatic filters further constrained lineage establishment (Elias et al. 2019; Marimon‐Junior et al. 2019).

### 2.2 Sampling of Occurrence Records

Occurrence records were compiled from vegetation inventory plots within the Cerrado biome (Alvarez et al. 2025). Only individuals with a base diameter > 5 cm at 30 cm above ground (Db_30cm_), sampled in plots unaffected by flooding or groundwater fluctuations, were included. Our analysis focused exclusively on savanna vegetation types of the Cerrado *stricto sensu* and its subdivisions: Cerrado *denso* (dense), Cerrado *típico* (typical), Cerrado *ralo* (sparse), and Cerrado *rupestre* (rocky outcrops) (Ribeiro and Walter 2008). To increase the representativeness of the Cerrado tree flora, we supplemented the dataset with publicly available records from 120 scientific publications. After correcting for typographical and taxonomic synonymy errors, 4006 occurrence records were retained, distributed heterogeneously across the biome and modeled species. From the Figshare platform, we provide the curated input data and computational code required to ensure the reproducibility of the analyses and figures (Alvarez 2025; https://doi.org/10.6084/m9.figshare.28020971), while raw records remain subject to the original licensing terms of the primary data providers.

Data for the 30 CHT were analyzed using two approaches. First, we considered each species independently. Second, following the concept of species combinations (Cáceres et al. 2012), we aggregated the 30 CHT and treated them as a single unit, termed a “mega‐species”. This approach characterizes the macro‐organization of the Cerrado and provides a broad synthesis representing all CHT. At the macroecological scale of this study, CHT exhibit similar soil‐climatic responses, with selectivity and preference for shared habitats (Alvarez et al. 2025).

### 2.3 Point‐To‐Point, Modeling and Evaluation

**FIGURE 1:**
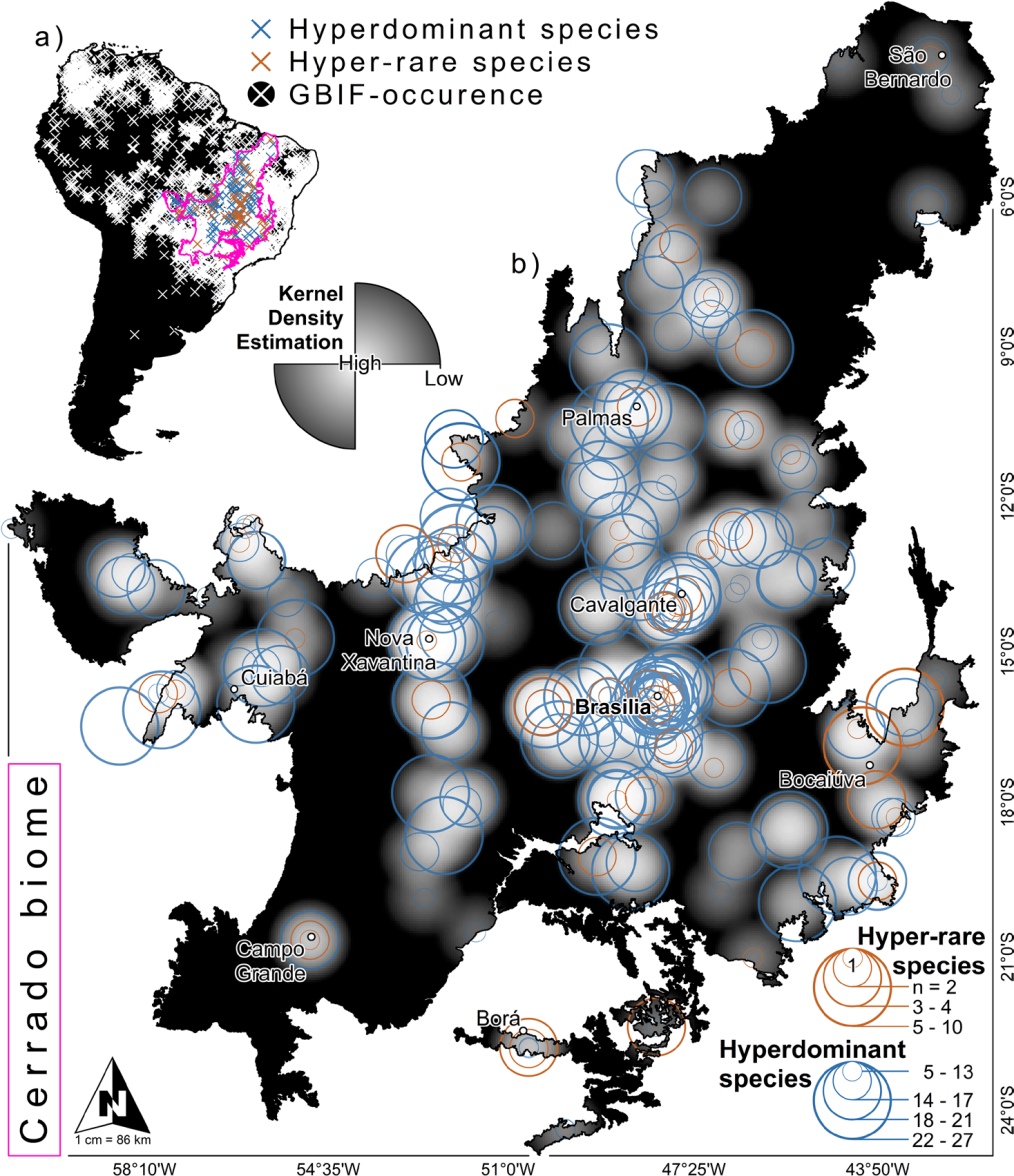
Distribution of hyperdominant and hyper‐rare species records. Occurrence records of hyperdominant (abundances > 50%, red crosses) and rare species (< 2 records, blue crosses), and occurrences obtained from the Global Biodiversity Information Facility (GBIF, white crosses) of the 30 Cerrado hyperdominant trees. The color gradients represent the maximum (blue and red colors) and minimum (lighter colors) variations in occurrence densities and the overlap between hyperdominant and rare species (violet color). Data and visual elements adapted from Alvarez et al. (2025).

Our SDMs estimate potential environmental suitability based on edaphoclimatic variables and do not explicitly constrain predictions to the realized niche. This approach allows us to identify (1) currently suitable but unoccupied habitat, which may reflect dispersal limitations or historical biogeographic barriers, and (2) areas projected to be suitable under future climate scenarios, recognizing that species' ability to colonize those areas will depend on dispersal capacity and other non‐climatic factors. To visualize the geographic distributions of plot‐level abundances and sampling efforts across the Cerrado (Figure 1), we performed a Kernel Density Estimation (KDE) using relative abundances in QGIS v.3.26.3 (QGIS.org 2020). For the current scenario, we used 19 bioclimatic variables from the WorldClim (https://worldclim.org). Future projections (2041–2060) were generated using three global circulation models (CCSM4, MPI‐ESM‐P, and MIROC‐ESM) under the Representative Concentration Pathway (RCP8.5), representing the highest greenhouse gas emissions scenario. This pathway was chosen as it represented the most pessimistic and plausible scenario of severe global climate change until 2100, considering the persistent rise in greenhouse gas emissions. Both current and future climate scenarios were complemented with six soil variables (bulk density, cation exchange capacity, coarse fragments, clay, sand, and silt) extracted from the SoilGrids platform (https://soilgrids.org). Complete details of the variable process, parameterization, and performance evaluation are provided in Appendix S1 (SM‐1: Modeling and evaluation).

### 2.4 Validation of Predictions and Conservation Status

To validate the predictions, we downloaded 12,684 occurrence records (OCCs‐GBIF) for the 30 CHT from the Global Biodiversity Information Facility (GBIF, https://doi.org/10.15468/dl.2mjn9a). The OCCs‐GBIF were independent of the occurrence records used as input data in the models (OCCs‐SDMs), and we removed overlapping records (buffer ≤ 1 km) to maintain data independence. Model performance was evaluated by quantifying the overlap between GBIF‐occurrence records and the modeled potential environmental suitability for each species in the current scenario. To estimate the model's performance and the correspondence of the predictions with the species' known presence, we calculated the percentage overlap value between the number of GBIF‐occurrence records and the geographic limits of the predictions obtained. We assessed the conservation status by overlaying the binary predictions (current and future) of the 30 CHTs onto the geographic boundaries of the protected areas reported by the IUCN for South America and the Cerrado biome. We extracted the protected areas from the World Database on Protected Areas Platform (WDPA: https://protectedplanet.net).

### 2.5 Functional Traits and Tree Hyperdominance

To evaluate the response of functional traits to climate change, we first created a functional traits database using previously published datasets (see Cruz et al. 2025). Traits were rescaled (mean = 0, SD = 1) and subjected to principal component analysis (PCA) across all species (17 species × 12 traits), using the ‘rda’ function from the *vegan* package (Oksanen et al. 2017). To reduce multicollinearity among environmental predictors, we calculated the variance inflation factor (VIF) using the ‘vifstep’ function in the *usdm* package (Naimi and Araújo 2016), retaining eight uncorrelated soil and climate variables. These variables were fitted to the first two PCA axes using ‘envifit’ and visualized with ‘ordiplot’. We analysed 12 functional traits Appendix S1 (SM‐2: Functional traits).

## 3 Results

### 3.1 Model Performance, Validation and Conservation Implications

The SDM captured the environmental distributions of the species across the Cerrado biome. Our model yielded high performance metrics, with Area Under the Curve (AUC) = 0.8 ± 0.003 (mean ± standard deviation), True Skill Statistic (TSS) = 0.7 ± 0.02, and Jaccard Index = 0.8 ± 0.01. Using KDE (Figure 1), we visualized the geographic distributions of species densities, identifying areas with three main patterns: (1) higher densities of hyperdominant species (in red); (2) higher densities of rare species (in blue); and (3) zones where both groups overlap. We defined these overlapping zones as species‐dominance cores (Alvarez et al. 2025), which largely coincide with climatically stable areas and known historical biodiversity refuges. The main species‐dominance core is concentrated in the central Cerrado, particularly around the Federal District. We also identified secondary dominance areas located on the plateaus and high plains of the Cerrado, characterized by shallow soils and marked seasonal rainfall patterns. These habitats have historically served as climatic refugia, associated with high densities of hyperdominant individuals. The KDE shows the distribution of sampling effort, highlighting geographic knowledge gaps and priority areas for planning future sampling (Figure 1).

**FIGURE 2:**
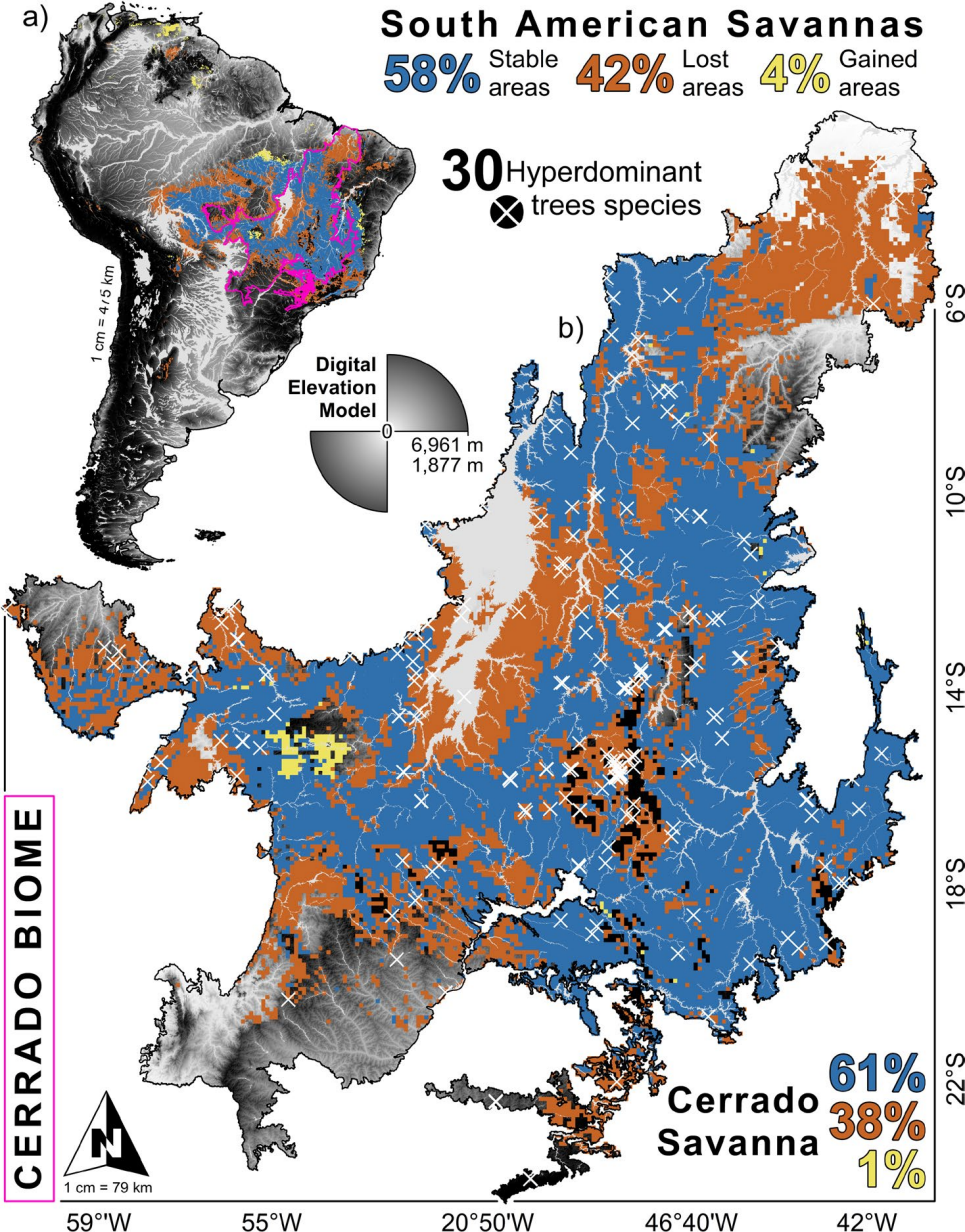
Model validation and conservation status of savanna physiognomies. (A) Occurrence records used to configure the algorithms (OCCs‐SDMs: Blue crosses) and occurrence records extracted from the Global Biodiversity Information Facility (OCCs‐GBIF: Green crosses) superimposed on potential environmental suitability (greyscale). (B) Protected areas from the World Database on the Protected Areas Platform superimposed on current binary predictions (green polygons) and future scenarios (blue polygons). Values were rounded for display; exact figures are provided in Table 1.

Validation of current predictions with GBIF occurrence records yielded an average agreement of 64% for all species. The potential environmental suitability is broadly consistent with known geographical occurrences, while also identifying environmentally suitable areas beyond current species' ranges, patterns that are congruent with established biogeographic barriers (e.g., *Qualea parviflora* and *Q. grandiflora* predicted north of the Amazon River). Out of 12,684 GBIF‐occurrence records for the 30 hyperdominant species, 6309 (49.7%) overlap with the potential environmental suitability, while 196 of 222 plot‐based records used to train the models (88.3%) fall within predicted suitable areas (Table 1, Figure 2). The highest agreement was observed for *Caryocar brasiliense* (90%), *Dalbergia miscolobium* (85%) and *Qualea parviflora* (81%). In contrast, the lowest agreements were found for *Xylopia aromatica* (18%), *Byrsonima coccolobifolia* (24%) and *Roupala montana* (26%) (Figure S1).

**TABLE 1:** Input data and spatial outputs of species distribution models (SDMs). Occurrence records used to train SDMs (OCCs‐SDMs) and those obtained from the Global Biodiversity Information Facility (GBIF) for model validation: Percentage of occurrence records overlapping the binary predictions (where 1 indicates predicted presence and 0 predicted absence). Values of potential environmental suitability (suitable area) and overlap of the predictions with protected areas. Conservation status according to the categories established in the International Union for the Conservation of Nature (IUCN): Not Evaluated (NE) and Least Concern (LC).

Hyperdominant species (in dominance order)	IUCN	OCCs‐SDMs	OCCs‐GBIF	Validation	Suitable area (%)			Current suitable area (km^2^)	Future suitable area (km^2^)	Protected areas in South America			
				OOCs—%	Stability	Loss	Gain			In current suitable area (km^2^)	(%)	In future suitable area (km^2^)	(%)
*Qualea parviflora*	NE	198	336	272%–81%	53.3	43.5	3.2	2,712,923	1,212,092	565,413	20.8	197,093	16.3
*Qualea grandiflora*	LC	200	373	300%–80%	57.8	38.5	3.7	3,102,416	1,557,489	647,748	20.9	287,928	18.5
*Curatella americana*	LC	118	764	264%–35%	52.7	44.1	3.2	2,612,763	1,157,448	557,034	21.3	185,314	16
*Pouteria ramiflora*	LC	170	345	221%–64%	47.2	50.5	2.3	2,405,692	918,373	467,170	19.4	141,961	15.5
*Tachigali vulgaris*	LC	133	147	79%–54%	46.9	50.8	2.3	2,117,782	767,128	357,998	16.9	91,564	11.9
*Davilla elliptica*	NE	135	437	345%–79%	44.2	53.0	2.8	2,114,344	695,536	409,245	19.4	90,957	13.1
*Hirtella ciliata*	LC	29	174	76%–44%	24.2	73.0	2.8	771,037	87,593	115,851	15	12,144	13.9
*Ouratea hexasperma*	NE	134	198	140%–71%	44.7	52.7	2.6	2,536,906	870,607	497,530	19.6	102,979	11.8
*Byrsonima coccolobifolia*	LC	181	617	349%–57%	46.1	52.3	1.6	2,309,802	1,106,481	479,071	20.7	218,588	19.8
*Byrsonima pachyphylla*	LC	120	276	218%–79%	39.3	58.2	2.5	1,701,267	462,388	297,886	17.5	43,596	9.4
*Kielmeyera coriacea*	NE	154	301	236%–78%	54.6	41.1	4.3	2,248,760	1,099,727	455,148	20.2	195,150	17.7
*Salvertia convallariodora*	LC	132	90	66%–73%	42.5	55.2	2.3	2,134,611	645,160	401,473	18.8	91,807	14.2
*Vatairea macrocarpa*	LC	141	69	50%–72%	52.0	46.8	1.2	2,392,653	974,794	452,598	18.9	123,381	12.7
*Lafoensia pacari*	LC	165	326	214%–66%	54.9	41.1	4.0	2,830,627	1,456,857	594,802	21	287,200	19.7
*Hymenaea stigonocarpa*	LC	170	345	247%–72%	54.9	42.0	3.1	2,530,032	1,188,385	527,525	20.9	218,709	18.4
*Byrsonima crassifolia*	LC	71	1452	345%–24%	46.1	52.3	1.6	2,605,771	864,443	524,489	20.1	110,144	12.7
*Plathymenia reticulata*	LC	126	383	239%–62%	50.2	47.3	2.5	2,437,460	963,652	489,636	20.1	144,025	14.9
*Eugenia dysenterica*	LC	99	184	145%–79%	53.3	42.4	4.3	2,523,750	1,198,816	536,997	21.3	183,006	15.3
*Connarus suberosus*	LC	165	278	219%–79%	48.5	48.4	3.1	2,271,042	865,391	384,836	16.9	108,808	12.6
*Bowdichia virgilioides*	LC	167	1002	435%–43%	52.6	44.7	2.7	2,587,875	1,135,165	506,880	19.6	209,844	18.5
*Caryocar brasiliense*	LC	141	192	173%–90%	60.1	33.0	6.9	2,540,942	1,487,558	511,496	20.1	306,023	20.6
*Roupala montana*	LC	159	781	204%–26%	56.2	38.4	5.4	2,560,852	1,351,603	543,434	21.2	276,878	20.5
*Xylopia aromatica*	LC	115	1295	235%–18%	50.6	45.4	4.0	2,172,308	970,171	400,016	18.4	141,232	14.6
*Qualea multiflora*	LC	135	263	200%–76%	39.7	53.9	6.4	1,843,389	575,109	286,107	15.5	83,428	14.5
*Aspidosperma tomentosum*	LC	137	632	482%–76%	49.3	46.9	3.8	1,930,153	742,950	331,768	17.2	90,957	12.2
*Terminalia argentea*	LC	76	179	94%–53%	52.8	42.3	4.9	1,968,435	903,439	357,512	18.2	119,616	13.2
*Astronium fraxinifolium*	LC	91	397	234%–59%	50.3	47.6	2.1	2,481,787	985,579	529,832	21.3	145,239	14.7
*Dalbergia miscolobium*	LC	107	303	259%–85%	55.6	38.2	6.2	2,544,021	1,374,600	582,172	22.9	199,401	14.5
*Erythroxylum suberosum*	LC	122	408	279%–68%	51.1	42.9	6.0	2,159,037	1,106,485	430,618	19.9	185,557	16.8
*Stryphnodendron adstringens*	NE	115	137	111%–81%	50.4	46.2	3.4	2,595,820	1,097,120	525,339	20.2	170,863	15.6

Predicted potential environmental suitability overlap with protected areas remained low across both climate scenarios (Figure S2). Currently, ~17.4% (~427,980 km^2^) of the estimated 2,462,350 km^2^ of suitable area lies within protected areas. Under future conservation‐oriented scenarios, ~18.1% (~231,377 km^2^) of the predicted 1,280,602 km^2^ is expected to remain within protected areas (Figure 2). Although the proportion within protected areas increases slightly, the absolute protected suitable area is projected to decline by ~45.9% (from ~427,980 to ~231,377 km^2^), reflecting an overall ~48.0% contraction in total extent (2,462,350 → 1,280,602 km^2^; current area ~1.92× the future area). In the current scenario, *Dalbergia miscolobium* (582,172 km^2^; 22.9%) and *Astronium fraxinifolium* (529,832 km^2^; 21.3%) exhibit the highest overlap with existing protected areas, while *Caryocar brasiliense* (511,496 km^2^; 20.6%) and *Roupala montana* (543,434 km^2^; 20.5%) are projected to retain the greatest coverage under future conditions. Species with low representation within protected areas, and thus greater vulnerability, include *Hirtela ciliata* (115,851 km^2^; 15%), *Qualea multiflora* (286,107 km^2^; 15.5%), *Byrsonima pachyphylla* (297,886 km^2^; 9.4%), *Ouratea hexasperma* (497,530 km^2^; 11.8%), and *Tachigali vulgaris* (357,998 km^2^; 11.9%).

### 3.2 Present and Future of the Cerrado Biome and South American Savannas

**FIGURE 3:**
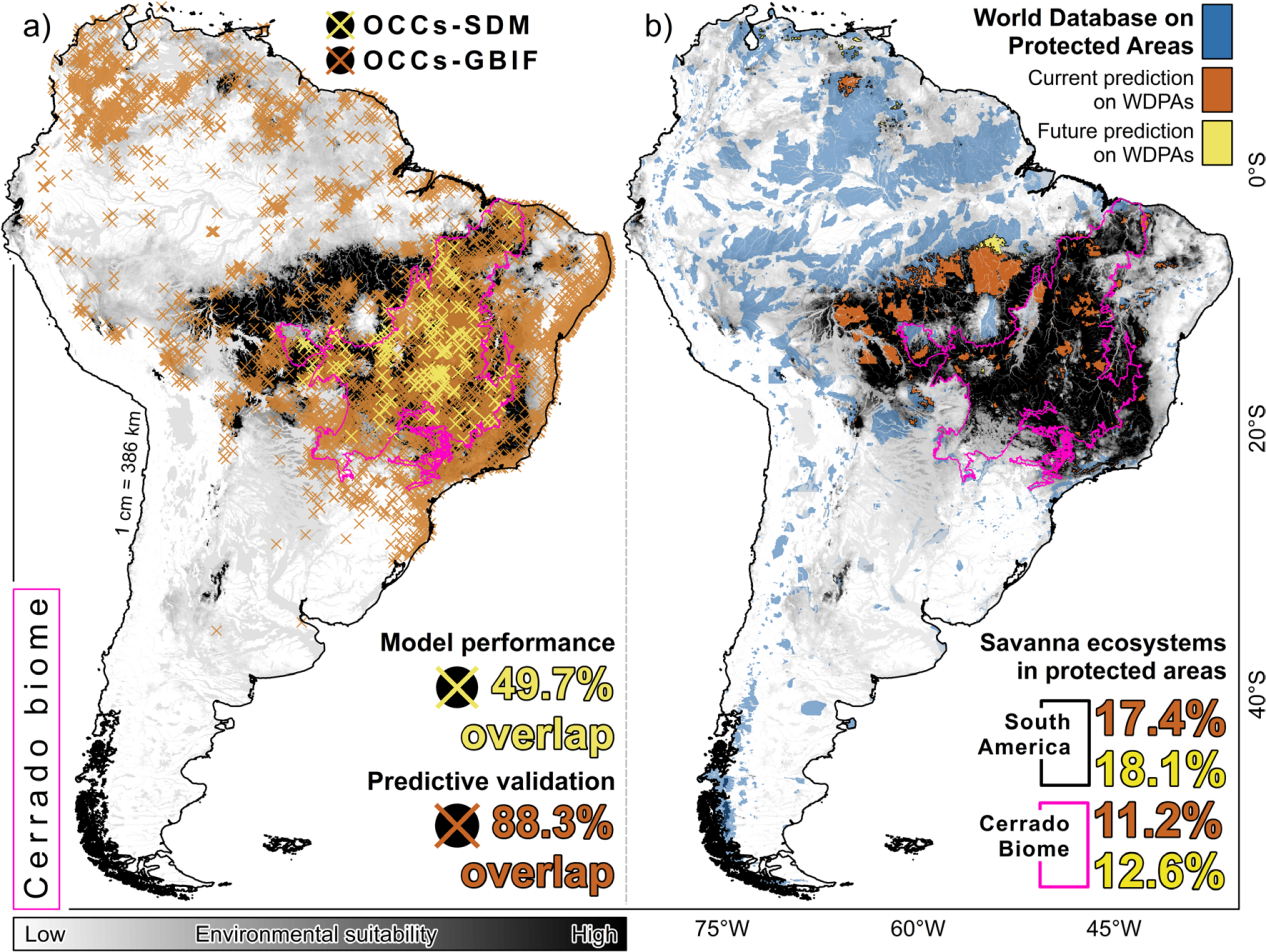
Transition of Cerrado hyperdominant trees under future climate scenarios. Potential environmental suitability for the 30 Cerrado hyperdominant trees (white crosses) in the current and future scenarios with the proportions of stability (blue color), loss (red color), and gain (green color) of potential suitability for the South American savannas and the Cerrado biome.

Current potential environmental suitability closely matches the known distribution of South American savannas. At the continental scale, our binary predictions for the mega‐species under the current scenario closely match the biogeographic regions where South American savanna physiognomies are recorded, particularly in Guyana, Venezuela, Bolivia, Brazil and Argentina (Figure 3). Specifically, the predicted suitable areas form a continuous patch of potential environmental suitability located between 5°–20° S and 50°–70° W. This predicted area overlaps key topographic formations, including the Brazilian Central Plateau and the northern Andes Mountains (Bolivia, Peru, Ecuador and Venezuela; Figure 3).

The set of projections used (CCSM4, MPI‐ESM‐P, MIROC‐ESM) suggests a widespread increase in mean annual temperature and a reduction or redistribution of seasonal precipitation across much of the Cerrado region. In particular, the projections show temperature increases concentrated in the dry season and changes in precipitation during the warmest quarter (bio18), implying greater water stress during critical growing months and a higher probability of conditions favorable for wildfires. Under projections of climate scenarios to ~2100, 58% of the potential environmental suitability for South American savanna is expected to remain stable. In contrast, 42% of the currently suitable area is expected to be converted to pasture and cropland. Only 4% of the continent could potentially host novel suitable habitat, depending on future environmental change and species dispersal capacity (Figure 3).

At the Cerrado biome scale, we identified two major areas of potential environmental suitability. The first spans 8°–20° S, 58°–65° W, overlapping the Serra dos Pacaás Novos (Rondônia) and the Aripuanã Park (Rondônia–Mato Grosso). The second covers 6°–17° S, 44°–53° W, encompassing the Central Plateau and the Serra Geral do Tocantins (Figure 3). These formations correspond to plateaus and ridges of quartzitic/gneissic nature, regions where topographic heterogeneity and seasonal rainfall create microrefuges with greater edaphoclimatic variability, favoring the persistence and diversification of endemic species. The predictions identified large patches of climatically suitable area in regions outside the species' known range, such as the Gran Sabana north of the Amazon River Basin. Under projected climate change, 61% of the current potential environmental suitability of the Cerrado is expected to remain stable, while 38% is likely to be lost due to land use conversion, and only 1% may become a suitable area for expansion in the central‐western region (Figure 3).

### 3.3 Cerrado Hyperdominant Trees Show Consistent, Convergent Suitable Areas

SDMs indicated that (1) potential environmental suitability for individual CHT is largely confined between 3°–23° S and 40°–74° W; (2) the average extent of environmental suitability is estimated at ~2,324,809 km^2^ under current climate conditions and ~994,071 km^2^ under future climate scenarios; (3) analysis of transition of suitable areas between current and future scenarios showed that individual CHT, on average, gained ~22,153 km^2^, retained ~920,927 km^2^, and lost ~496,855 km^2^ of suitable area; (4) currently, only 17.4% of South American savanna ecosystems (11.2% in the Cerrado) are contained within protected areas, a proportion projected to fall to 18.1% (12.6% in the Cerrado) under future scenarios; (5) although climatically stable areas remain concentrated in the central region, both losses and gains in potential suitability are primarily located along the boundaries of the predicted South American savanna distribution; and (6) at the continental scale, areas of low potential suitability are mainly concentrated in two quadrants: a northern sector (6°–11° S, 50°–79° W) and a southern sector (23°–54° S, 22°–48° W; Figure S3).

The top three hyperdominant species show the greatest suitable area stability under future climate scenarios. However, this trend diminishes from the fourth species onward, with a loss of suitability among lower‐ranking hyperdominants. Of the 30 species analyzed, 17 are projected to maintain stable suitability, nine are expected to experience contractions in suitability, and only four may gain new suitable areas under future climate conditions. *Hirtella ciliata* (7th in dominance rank) is projected to lose 73% of its suitable area, followed by *Byrsonima pachyphylla* (10th: 58%) and *Salvertia convallariodora* (12th: 55%), making them the species with the largest reduction in potential environmental suitability. In contrast, *Caryocar brasiliense* (21st in dominance rank: 60% stability of suitable areas), *Qualea grandiflora* (2nd: 58%), and *Roupala montana* (22nd: 56%) are predicted to retain most of their current suitable areas across spatial–temporal predictions. The most favorable suitable‐area gain scenarios are associated with *Caryocar brasiliense* (21st in dominance rank: 7% suitable‐area loss), *Qualea multiflora* (24th: 6%), and *Dalbergia miscolobium* (28th: 6%) (Figure S3). Together, *D. miscolobium*, *C. brasiliense* , *R. montana* , and *Q. grandiflora* stand out as the species with the highest overall stability of suitable areas, exhibiting the highest potential for suitable‐area expansion and the lowest projected losses of suitable area under future climate conditions, without considering other ecological or socioeconomic factors.

### 3.4 Functional Traits, Current and Future of the Cerrado Biome

**FIGURE 4:**
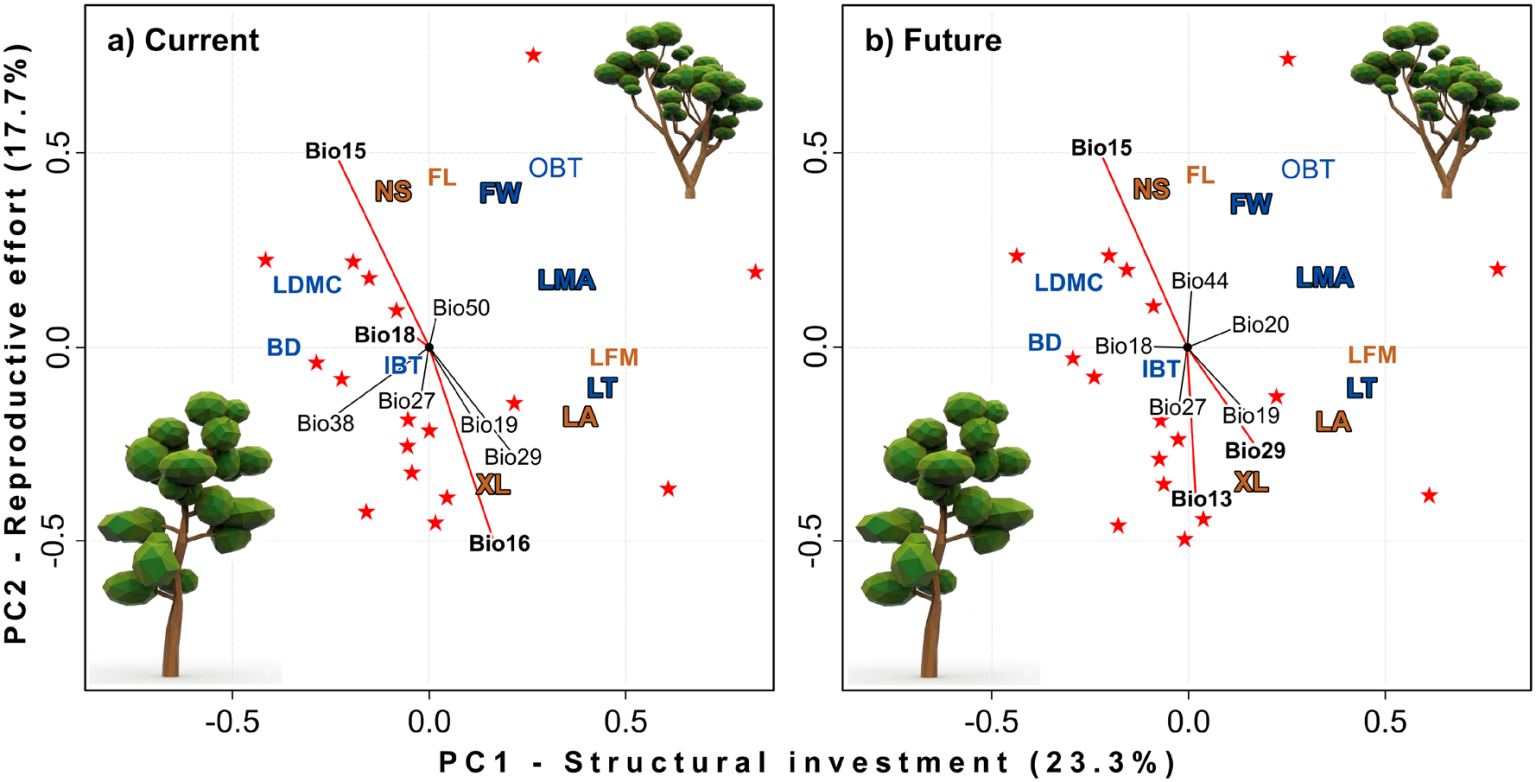
PCA of trait–environment relationships for 30 hyperdominant species. The first axis (PC1, 23.3% of variance) describes a gradient of structural investment where blue labels identify traits with the highest loadings, such as leaf thickness (LT), leaf mass per area (LMA), and fruit width (FW). Variation along the second axis (PC2, 17.7%) is primarily driven by reproductive effort and organ size, represented by brown labels including number of seeds (NS), leaf area (LA), and xylem length (XL). Bolded vectors denote the most influential environmental predictors identified for each period. In the current scenario, precipitation seasonality (bio15), precipitation of the wettest quarter (bio16), and precipitation of the warmest quarter (bio18) emerge as the dominant filters for functional strategies. Under future conditions, the model highlights a shift in environmental pressure where precipitation seasonality (bio15), absolute depth to bedrock (bio29), and precipitation of the wettest month (bio13) become the primary determinants of trait distribution. The vegetation sketches illustrate the transition from acquisitive species with large, thin leaves toward conservative species that prioritize investment in protective tissues.

The principal component analysis (PCA) highlighted distinct ecological strategies and gradients among hyperdominant tree species (Table S1). Principal Component 1 (PC1) captured the main trait axis, separating “fast” species (acquisitive strategies: high photosynthesis, larger and less dense leaves) and “slow” species (conservative strategies: thicker and denser leaves in limiting environments). The PC2 reflected gradients linked to microclimatic and edaphic variation, capturing ecological divergence potentially related to speciation processes (Table S1, Figure 4).

Under current climate conditions, PC1 was driven primarily by precipitation in the coldest quarter (bio19, CV = 0.523) and depth to bedrock (bio29, CV = 0.538), highlighting their importance in structuring species' functional differentiation (Table S1, Figure 4). Temperature variables (bio5 and bio10) were positively associated with fast‐species growth strategies, while precipitation variables (bio18 and bio19) correlated with slow or environmentally specialized strategies. Functional traits aligned closely with this gradient: species with thicker leaves, higher dry mass, and greater leaf mass per area were positioned along the “slow strategies” end of PC1, while species with larger leaf area and higher fresh mass were associated with “fast” strategies. Traits such as inner bark thickness (IBT) and outer bark thickness (OBT) reflected intermediate strategies, with IBT notably linked to species occurring on shallow, rocky soils (e.g., bio38; Table S1, Figure 4). PC1 was also influenced by traits such as leaf fresh mass (LFM, CV = 0.473) and fruit width (FW, CV = 0.473), whereas PC2 was primarily defined by flower length (FL, CV = 0.443) and number of seeds (NS, CV = 0.401). Among environmental variables, silt content (bio50, CV = 0.982) and seasonal precipitation (bio15, CV = 0.902) showed strong positive correlations in PC2. Notably, maximum xylem length exhibited consistent associations with multiple precipitation‐related variables (bio15, bio16, bio19, and bio29), underscoring its ecological importance in seasonal environments. Overall, traits related to water‐use efficiency showed strong interactions with environmental gradients under both current and future scenarios (Table S1, Figure 4).

In the future climate scenario, PC1 was strongly influenced by precipitation in the warmest quarter (bio18, CV = −0.994) and saturated soil water content (bio20, CV = 0.920). Notably, bio18 has little effect under current conditions, indicating a climate‐driven shift in environmental filtering (Table S1, Figure 4). PC2 remained dominated by silt content (bio44, CV = 0.997) and seasonal precipitation (bio15, CV = 0.913), similar to patterns observed in the current scenario. Overall trait–environment relationships remained qualitatively consistent across time periods, but the magnitude of their correlations changed between Current‐Future. For instance, LFM and FW remained influential in PC1, while FL and NS lost relevance in PC2 under future conditions. Future projections emphasized reproductive traits, particularly seed number and fruit size (width and length) for species success. These traits were primarily associated with negative correlations with bio13 (precipitation of wettest month) and bio27 (depth to bedrock), and a positive correlation with bio44 (Table S1, Figure 4). While depth to bedrock and silt content remained important across both scenarios, their relative contributions shifted, silt content not included among the set of variables in the future model. In contrast, precipitation variables, specifically bio18 (warmest quarter precipitation), increased in importance in the future and showed a pronounced negative loading on PC1 (CV = −0.994), indicating that higher bio18 values are associated with the portion of the gradient less favorable for fast‐strategy species under the RCP8.5 scenario.

## 4 Discussion

### 4.1 Cerrado Hyperdominant Trees Act as Proxies for South American Savannas

The CHTs function as biogeographical and climatic indicators of the South American savannas because: (i) their distributions synthesize historical patterns of climatic stability, (ii) they point to areas with edaphoclimatic conditions appropriate for the persistence of the savanna physiognomy, and (iii) they allow the identification of regions with future climatic vulnerability that can anticipate changes in the structure and functioning of the ecosystem (Werneck et al. 2012; Bueno et al. 2018; Françoso et al. 2020). Paleoecological evidence suggests that forests had a patchy distribution in humid climate refugia across the Amazon Basin during the Last Glacial Maximum (LGM; 20–13 Ka; Bueno et al. 2017; Costa et al. 2018, 2022). The Pleistocene refugia hypothesis posits that this drier climate period, characterized by lower temperatures and precipitation, combined with more recent anthropogenic drivers such as megafauna extinction and the use of fire (Durigan 2020; Li et al. 2021), led to the expansion of the Cerrado and the contraction of Amazonian forests (Buzatti et al. 2018; Costa et al. 2018). Our predictions align with historical remnants of the South American savanna. These include the Amazon Dry Corridor, a discontinuous corridor of dry vegetation connecting savanna remnants in northern South America; the Caatinga, a semi‐arid biome in northeastern Brazil with xerophytic vegetation; and the Chaco, which comprises savannas and dry forests in Bolivia, Paraguay, and Argentina (Werneck et al. 2012; Buzatti et al. 2018). The convergence of our predictions with these regions supports the biogeographical validity of the models. Additionally, our predictions identified areas of potential environmental suitability in regions where some Cerrado species are not currently found, such as the savannas of Venezuela and Guyana. This discrepancy is expected, as the predictions map the fundamental niche based solely on environmental variables, whereas species distributions are also shaped by other ecological factors: geographical barriers, biotic interactions, fire regimes, anthropogenic disturbance, and current land use (Diniz‐Filho et al. 2019). For instance, *Qualea parviflora* and *Q. grandiflora* do not occur north of the Amazon River, despite the model predicting suitable conditions in those areas. The absence of these species from climatically suitable northern savannas highlights the role of the Amazon River Basin as a long‐standing biogeographic barrier to dispersal for Cerrado flora (Werneck et al. 2012; Bueno et al. 2018; Buzatti et al. 2018). No field records confirm their occurrence in these predicted areas; hence, while these northern regions are climatically analogous, they are unlikely to be naturally colonized and do not constitute part of the species' realized niche (Diniz‐Filho et al. 2019).

Cerrado tree hyperdominance is shaped by orogenic and paleoclimatic processes. While paleoclimatic cycles compressed and fragmented habitats, topographic heterogeneity created microrefuges with relatively stable conditions in the central Cerrado region (Costa et al. 2018, 2022; Pfeilsticker et al. 2021). Together, these factors favored the accumulation and persistence of diversity in the center of the biome, generating the observed center‐periphery pattern (Alvarez et al. 2025). During the Last Glacial Maximum (LGM), the highest elevations (> 800 m) in central Brazil experienced cold, semi‐arid conditions with strong winds, partial soil exposure, and high erosion (Bueno et al. 2017; Horák‐Terra et al. 2025). By contrast, interplateau depressions and wind‐sheltered slopes maintained warmer temperatures, deeper soils, and higher soil water storage (Horák‐Terra et al. 2025). Phylogeographic studies of three hyperdominant Cerrado tree species, such as *Caryocar brasiliense* , *Hymenaea stigonocarpa*, and *Plathymenia reticulata*, revealed higher genetic diversity in these central Cerrado depressions, consistent with LGM climate refugia (Buzatti et al. 2018; Costa et al. 2018; Pfeilsticker et al. 2021). Isotopic evidence from forest‐savanna replacement suggests that Holocene warming and increased humidity favored Cerrado expansions (Costa et al. 2022). This is further supported by the wide distribution of *Curatella americana* (5th in dominance rank), which currently occurs across the Cerrado, the Atlantic Forest, and the Amazon (Silvério et al. 2013; Bueno et al. 2017; Costa et al. 2018).

The historical and biogeographic context affecting Cerrado hyperdominance also has direct implications for its future resilience. Changes in potential environmental suitability (stability, loss, and gain) in the 30 CHT are crucial for regional conservation, as they directly reflect the ecosystem's resilience to climate change (Werneck et al. 2012; Costa et al. 2018). High stability of suitability indicates persistence in current ranges, whereas loss of suitable area, projected for 42% of current suitable area under future scenarios, signals habitat reduction requiring urgent conservation action. Conversely, limited gain in potential environmental suitability suggests few new suitable areas, but colonization will depend on dispersal capacity. The low representation of environmental suitability within protected areas, which decreases from 19.5% to 15.3% in future projections, highlights the need to align conservation priorities with long‐term suitable geographic areas (hyperdominant climate refuges), since the persistence of the functional integrity of the biome depends more on the protection of these refuges than on mere coincidence with the current distribution of individuals.

### 4.2 Spatial and Climatic Determinants of South American Savanna Distribution

Latitudinal and altitudinal corridors connect South American highland savannas. Suitability maps overlapped with the tabular quartzite mountains of the Guiana Shield (Tepuyes) and with Pico da Neblina, Brazil's highest peak (Werneck et al. 2012; Buzatti et al. 2018). In line with floristic records of high‐altitude savanna formations (Cerrado rupestre), suitable areas extended along granite‐gneissic mountains of the Brazilian east coast. The predictions also revealed a continuous latitudinal corridor linking the Brazilian Cerrado to the savannas of the Bolivian and Argentine Chaco via the Sub‐Andean ranges.

Macroecological patterns derived from CHT‐based predictions indicate that potential environmental suitability for South American savannas is constrained north and south by the Intertropical Convergence Zone, a likely climatic boundary (Bueno et al. 2018). Paleoclimatic fluctuations appear to have facilitated bidirectional colonization between adjacent biomes, particularly across the complex and megadiverse Cerrado‐Amazon transition zone (Bueno et al. 2017; Costa et al. 2022). Our suitability predictions align with northern Amazonian savannas, such as Sipaliwini‐Parú, and in the south, suitable areas extend into parts of Mato Grosso and Rondônia. In the Cerrado‐Caatinga transition, suitability coincides with the Araripe Plateau, which shares stronger floristic affinities with the core Cerrado than with the Caatinga (Bueno et al. 2018).

### 4.3 Cerrado Trees Coevolved With Extreme Drought and Fire Events

The increase in temperature, combined with greater seasonal variations in precipitation and reduced fuel moisture, can increase the frequency and extent of fires (see Durigan 2020; Li et al. 2021). Climate change and insolation do not appear to affect species' environmental and geographic fidelity (Silvério et al. 2013; Bueno et al. 2017; Durigan 2020; Hofmann et al. 2023). However, climate change, together with recurrent fire events and the advancing Arc of Deforestation, may create “empty niches” across the biome (Diniz‐Filho et al. 2020; Li et al. 2021; Hofmann et al. 2023). Our suitability predictions for CHT are consistent with Pfeilsticker et al. (2021), who identified *Qualea grandiflora* (2nd in dominance rank) as highly suitable in the central Cerrado, coinciding with the distribution of several typical and endemic species (Buzatti et al. 2018; Costa et al. 2022). These endemism centers correspond to Quaternary climatic stability Pfeilsticker et al. (2021), using SDMs, confirmed long‐term climatically stable Pleistocene refugia in the central Cerrado, findings supported by complementary paleoecological and genetic studies (Werneck et al. 2012; Bueno et al. 2017; Buzatti et al. 2018; Costa et al. 2018, 2022; Overbeck et al. 2022). While our future predictions geographically coincide with the climate stability findings of Pfeilsticker et al. (2021), we have evidenced contrasting variations in the spatial patterns of our target species. For example, *Stryphnodendron adstringens* (23rd) may gain environmental suitability, potentially due to its high leaf phenols and flavonoid concentrations and allelopathic effects (Silva et al. 2006). In contrast, *Plathymenia reticulata* (26th) may face reduced suitability, possibly due to low genetic diversity (Lacerda et al. 2001), especially in northeastern and southern Brazilian Cerrado populations, and historical population bottlenecks associated with the drier and colder LGM climate (Novaes et al. 2010).

### 4.4 The Resilience of Savannas Hinges on How Functional Traits Respond to Climate Change

Leaf biomass and fruit width emerged as key traits (predictors of persistence under RCP8.5), suggesting that traits related to biomass accumulation and structural investment support adaptation to future climates (Li et al. 2021; Souza et al. 2022). Our predictions indicate that traits linked to biomass production and reproductive strategies will be central for selecting and maintaining resilient Cerrado species (Pfeilsticker et al. 2021; Cruz et al. 2025). This pattern is consistent with empirical evidence showing that savanna lineages (open vegetation types) often exhibit functional strategies such as rapid growth, higher reproductive investment, and disturbance tolerance, in contrast to forest species of the closed and dense vegetation types of the Cerrado. However, we recognize that there is overlap in strategies and that these characteristics are not mutually exclusive. The Cerrado is becoming, on average, warmer and drier, decreasing the functional performance of the 30 CHT; reductions in rainfall frequency lengthen drought periods, reduce water availability, and increase the likelihood of extreme fires (Hofmann et al. 2023). The survival of these species will also depend on soil properties, mainly the volumetric content of saturated water and the silt content, which together control water and nutrient retention in seasonally dry climates (Elias et al. 2019; Marimon‐Junior et al. 2019). Greater climatic variability and reliance on precipitation and soil water may disadvantage fast‐growing species poorly adapted to arid environments (Marimon‐Junior et al. 2019). The projections used mainly indicate a seasonal redistribution (variation in bio15, bio18, and bio19) rather than a uniform loss of precipitation, which increases interannual variability and seasonal stress during growth periods, affecting the distribution of species (Ribeiro‐Júnior et al. 2023). In arid regions, hydrology and soils remain decisive, with competition mediated by trade‐offs between functional traits and climate tolerance (Souza et al. 2022; Ribeiro‐Júnior et al. 2023; Cruz et al. 2025). Species relying on animal or water dispersal may be more vulnerable to environmental change than wind‐dispersed species (Diniz‐Filho et al. 2020; Ribeiro‐Júnior et al. 2023).

### 4.5 Conservation Planning Should Prioritize Centers of Endemism

About 58% of the remaining native vegetation of the Cerrado biome is on private lands and outside protected areas (Alencar et al. 2020; de Oliveira et al. 2025), while only ~8% of the biome is legally protected (Françoso et al. 2020), highlighting a major protection gap. Since 44% of Brazil is privately owned and 58% of the Cerrado's native vegetation remains on such lands, responsibility for its future falls primarily on national governance and private stakeholders (Strassburg et al. 2017; Colman et al. 2022; de Oliveira et al. 2025). Our predictions overlap climatically stable refugia, areas that historically and currently hold the highest genetic diversity, species richness, abundance and endemism, and many of these refugia lie outside protected areas (Myers et al. 2000; Buzatti et al. 2018; Diniz‐Filho et al. 2020). Although effective conservation has been reported in the Federal District and Tocantins (Bueno et al. 2017), climatically stable refugia in Minas Gerais, Bahia, Maranhão, Piauí, Mato Grosso do Sul and São Paulo states remain unprotected.

Strategic conservation planning must therefore be regionally tailored. Priority actions include expanding protected areas in the central region, northwest and central‐west, prioritizing the MATOPIBA states (an acronym linking portions of the states of Maranhão, Tocantins, Piauí, and Bahia, representing an agricultural frontier) where agricultural and ranching expansion is ongoing, and establishing new protected areas in the northeast, northwest and southwest. Complementary measures, private reserves and incentives for natural regeneration, are best targeted to the central‐east, south and central‐west (Strassburg et al. 2017; Françoso et al. 2020; Overbeck et al. 2022; Colman et al. 2022). The Cerrado has experienced the greatest proportional native‐vegetation loss among Brazil's major biomes (~27% loss between 1985 and 2023; MapBio 2024), and projections indicate that by 2070 population growth and agricultural expansion could reduce remaining native vegetation to ~35% of its original extent, mostly within protected areas, changes that risk biome‐wide collapse of ecological persistence (Alencar et al. 2020; Vieira et al. 2022; Colman et al. 2022; de Oliveira et al. 2025).

In this research, we show that a small group of 30 species encapsulates the vulnerability of the Cerrado: their responses, combined with edaphoclimatic variables, determine both the stability and the projected contraction of environmental suitability at the biome scale. Our results indicate that most of the persistent climate suitability is concentrated in historical refuges and that current representation in protected areas is insufficient to guarantee the future persistence of the ecosystem's functional structure. The conservation of the Cerrado will require policies that integrate (i) the identification and protection of climate refuges, (ii) actions based on functional traits to prioritize vulnerable species and populations, including assisted migration where barriers may limit dispersal, and (iii) fire and land‐use management strategies that reduce negative synergies between climate change and habitat loss. The recent Senate approval of Bill No. 2.159/2021 (known as the “Devastation Bill”), which modifies the environmental licensing regime, increases the urgency of aligning land‐use governance with conservation priorities; without such alignment, the negative synergies between climate change and deregulated habitat loss risk weakening the functional integrity of this biodiversity hotspot.

## Author Contributions

**Beatriz Schwantes Marimon:** conceptualization, investigation, funding acquisition, writing – original draft, writing – review and editing, visualization, project administration, formal analysis, software, supervision, resources, data curation. **Ben Hur Marimon‐Junior:** supervision, resources, project administration, conceptualization, investigation, funding acquisition, writing – original draft, writing – review and editing, visualization. **Facundo Alvarez:** conceptualization, investigation, funding acquisition, writing – original draft, writing – review and editing, visualization, validation, methodology, software, project administration, formal analysis, data curation, supervision, resources. **Wesley Jonatar Alves da Cruz:** writing – original draft, writing – review and editing, software, formal analysis, data curation, resources, methodology, investigation. **Raiane Gonçalves Béu:** writing – review and editing, writing – original draft. **Marcelo Leandro Bueno:** writing – original draft, writing – review and editing, data curation, resources. **Polyanna da Conceição Bispo:** writing – review and editing, writing – original draft. **Bruno Machado Teles Walter:** writing – original draft, writing – review and editing, resources, data curation. **Ricardo Flores Haidar:** data curation, resources, writing – review and editing, writing – original draft. **Eddie Lenza de Oliveira:** writing – original draft, writing – review and editing, data curation, resources. **Edson de Souza Lima:** resources, data curation, writing – review and editing, writing – original draft. **Sabrina do Couto de Miranda:** writing – original draft, writing – review and editing, data curation, resources. **Oliver Phillips:** writing – original draft, writing – review and editing, data curation, resources, conceptualization, investigation, supervision. **Zenésio Finger:** writing – original draft, writing – review and editing, data curation, resources. **Paulo Sérgio Morandi:** data curation, resources. **Ted R. Feldpausch:** conceptualization, investigation, funding acquisition, writing – original draft, writing – review and editing, visualization, supervision, resources, project administration. **Norberto Gomes Ribeiro Júnior:** writing – original draft, writing – review and editing, software, formal analysis, data curation, resources, methodology, investigation. **Aldair de Souza Medeiros:** writing – review and editing, data curation, resources. **Glécio Machado Siqueira:** writing – review and editing, data curation, resources. **Fabiana de Gois Aquino:** writing – review and editing, data curation, resources. **Frederico Augusto Guimarães Guilherme:** writing – review and editing, data curation, resources. **José Roberto Rodrigues Pinto:** writing – review and editing, data curation, resources. **Henrique Augusto Mews:** writing – review and editing, data curation, resources. **Renata Dias Françoso Brandão:** writing – review and editing, data curation, resources. **Eraldo Aparecido Trondoli Matricardi:** writing – review and editing, data curation, resources. **Cássia Beatriz Rodrigues Munhoz:** writing – review and editing, data curation, resources. **Maria Antônia Carniello:** writing – review and editing, data curation, resources. **Mercedes Maria da Cunha Bustamante:** writing – review and editing, data curation, resources. **Edmar Almeida de Oliveira:** data curation, resources. **Eder Carvalho das Neves:** writing – review and editing, data curation, resources. **Fernando Elias:** writing – review and editing, data curation, resources. **Immaculada Oliveras Menor:** writing – review and editing, data curation, resources. **Simone Matias de Almeida Reis:** writing – review and editing, data curation, resources.

## Funding

This study was supported by the Coordenação de Aperfeiçoamento de Pessoal de Nivel Superior (CAPES PVE 2012‐177) and the UK Natural Environment Research Council (NERC) (NE/N011570/1; NE/W001691/1). M.L.B. was supported by a Conselho Nacional de Desenvolvimento Científico e Tecnológico (CNPq) productivity grant (PQ‐C, #313179/2022‐0). C.B.R.M. (#303556/2025‐0), F.A.G.G. (#302200/2025‐8), and J.R.R.P. (#312571/2021‐6) also receive research productivity fellowships from CNPq. This research was further supported by the Fundação de Amparo à Pesquisa do Estado de Goiás (FAPEG) through the PELD‐CEMA program (Processes #2017/10267000329 and #2021/10267000959).

## Conflicts of Interest

The authors declare no conflicts of interest.

## Supporting information


**Figure S1:** Cerrado hyperdominant validation.
**Figure S2:** Conservation status of the South American savanna ecosystem and the Cerrado biome.
**Figure S3:** Comparisons between current and future climate scenarios.

**Table S1:** PCA loadings for edaphoclimatic variables and functional traits.

**Appendix S1:** Supplementary Methods
SM‐1: Modeling and evaluation.
SM‐2: Functional traits.

## Data Availability

The data that support the findings of this study, including the processed occurrence records and computational code used for species distribution models and figures, are openly available in Figshare at https://doi.org/10.6084/m9.figshare.28020971 (Alvarez 2025). Secondary data sources for species occurrences and environmental predictors are cited within the manuscript and available from their respective public repositories (e.g., Alvarez et al. 2025).
