# Supplementary material for: Hyperdominant Trees Reveal Savanna Vulnerability Under Climate Change
Source: Glob Chang Biol. 2026 Apr 16;32(4):e70859. doi: 10.1111/gcb.70859 (PMC13087481; doi:10.1111/gcb.70859)
Supplement: Supplementary file 1 — Figure S1: Cerrado hyperdominant validation. Figure S2: Conservation status of the South American savanna ecosystem and the Cerrado biome. Figure S3: Comparisons between current and future climate scenarios. [file GCB-32-e70859-s001.docx]

**Supplementary Information - Supplementary Figures**


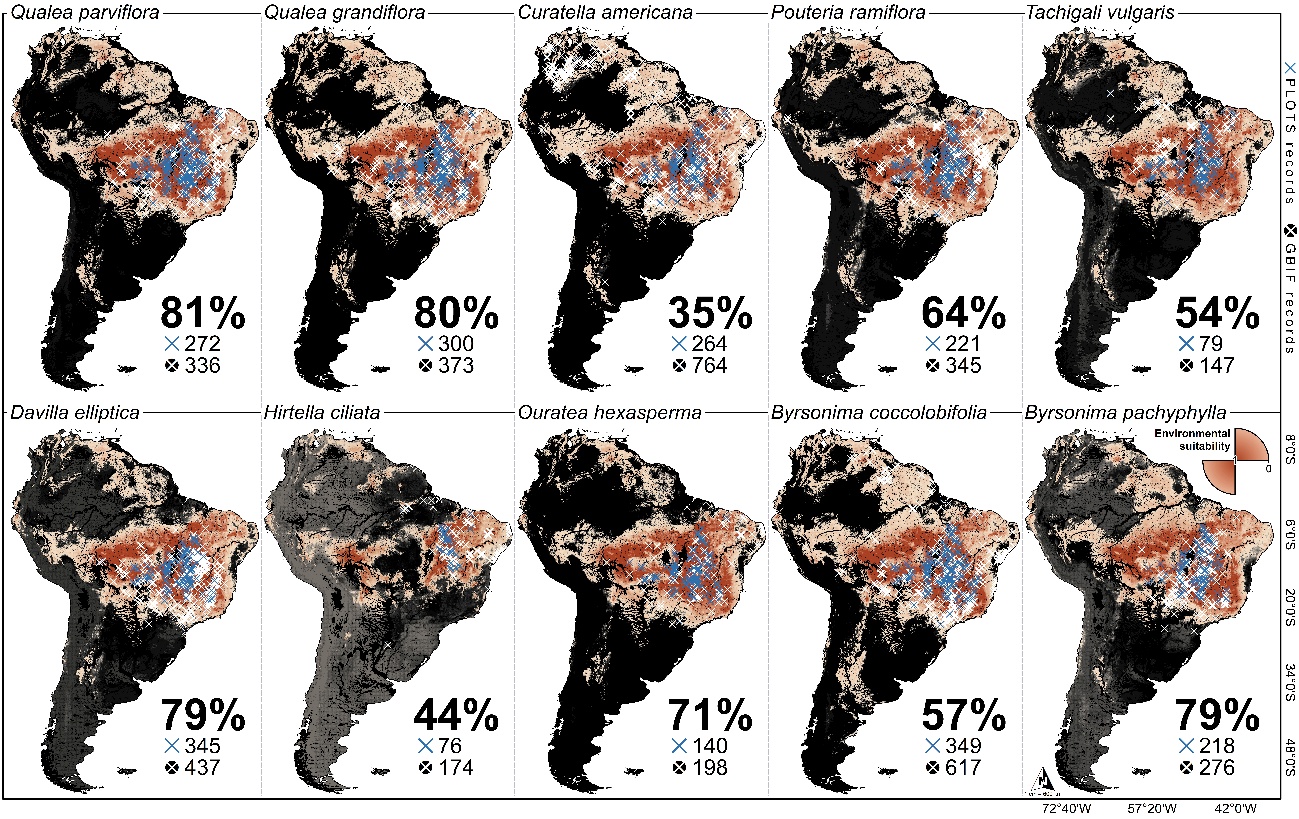


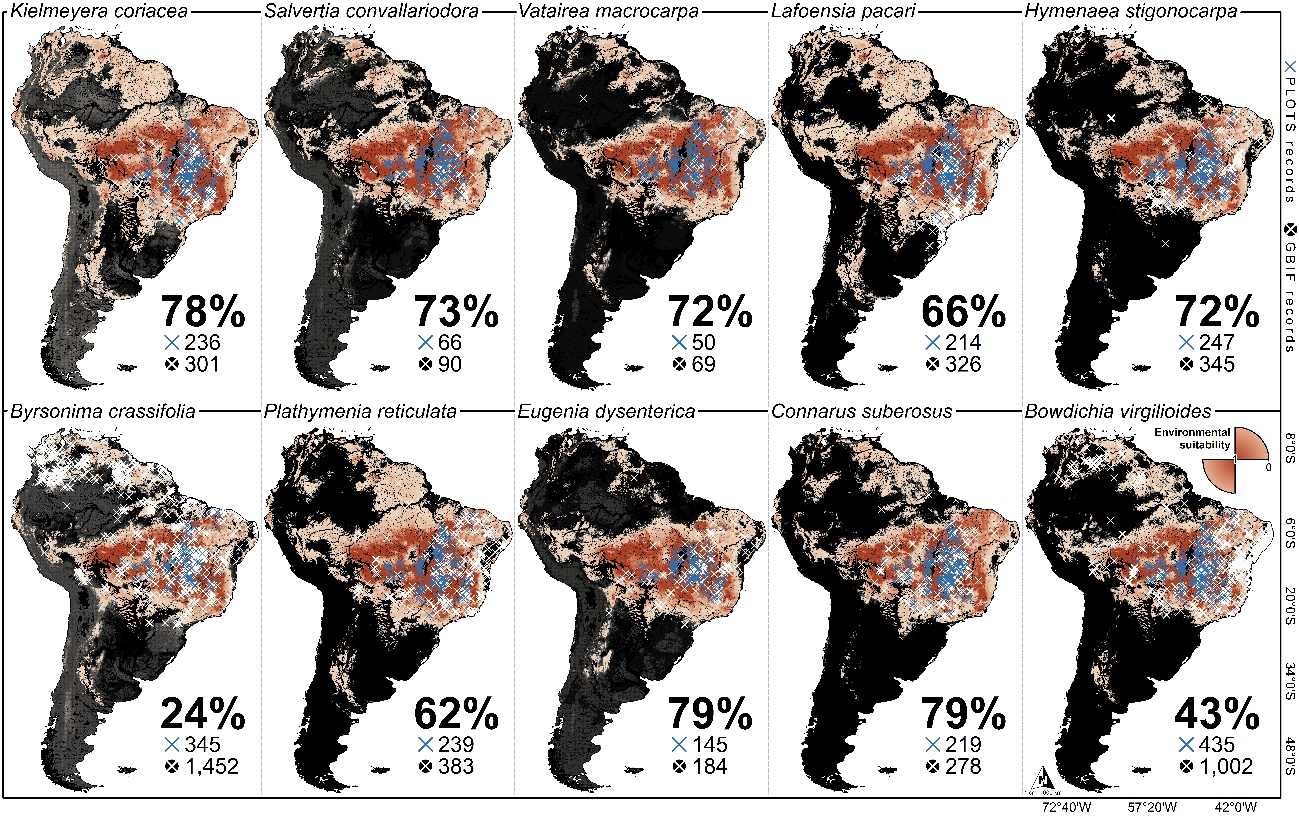


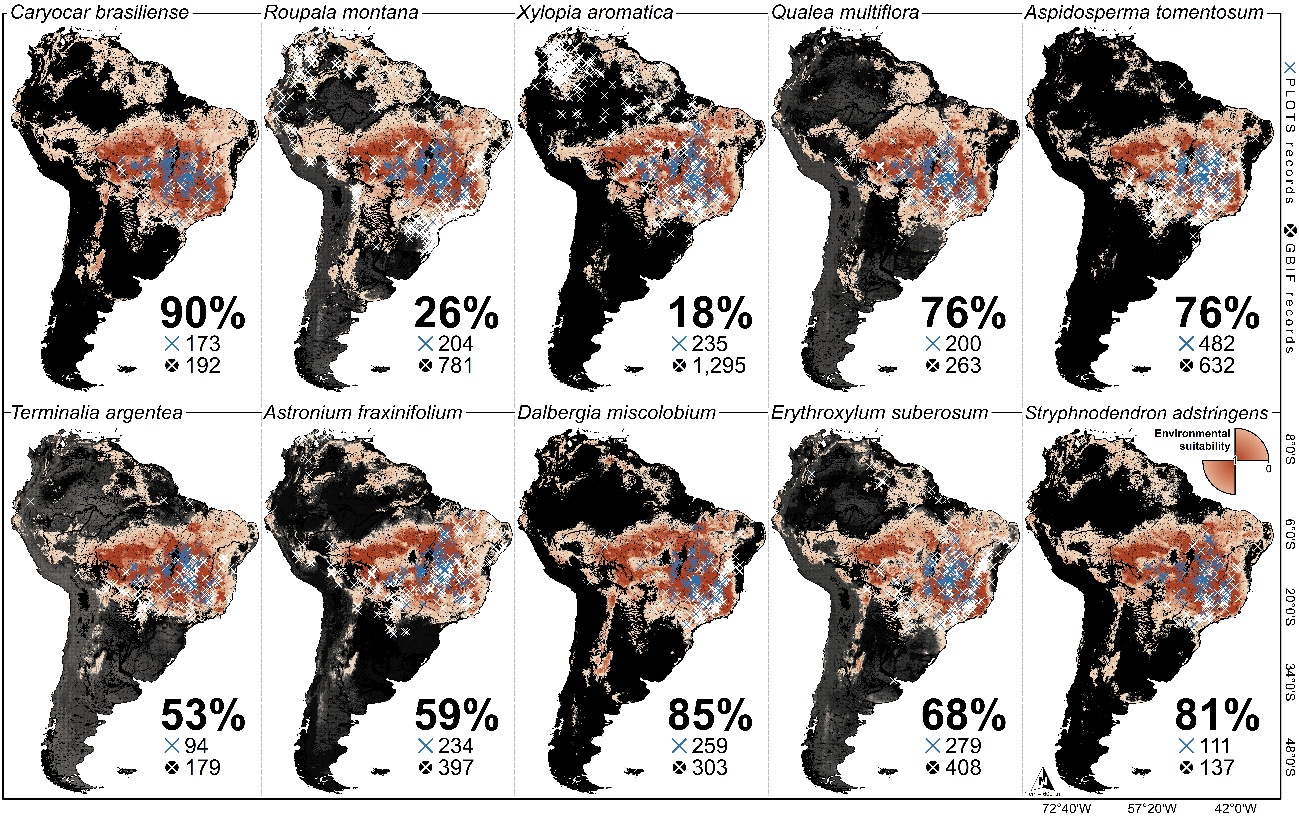


**Supplementary Figure 1: Cerrado hyperdominant validation.** Maps show the potential environmental suitability for each hyperdominant species in the Cerrado biome, ranging from grey/beige (low suitability) to orange/red (high suitability). Blue crosses indicate PLOTS records used for model calibration, while white crosses represent Global Biodiversity Information Facility (GBIF) records used for external validation. Percentage values indicate the proportion of GBIF records overlapping with the binary predictions of potential suitability. Values displayed on the maps are rounded for visual clarity; exact values are reported in Table 1.


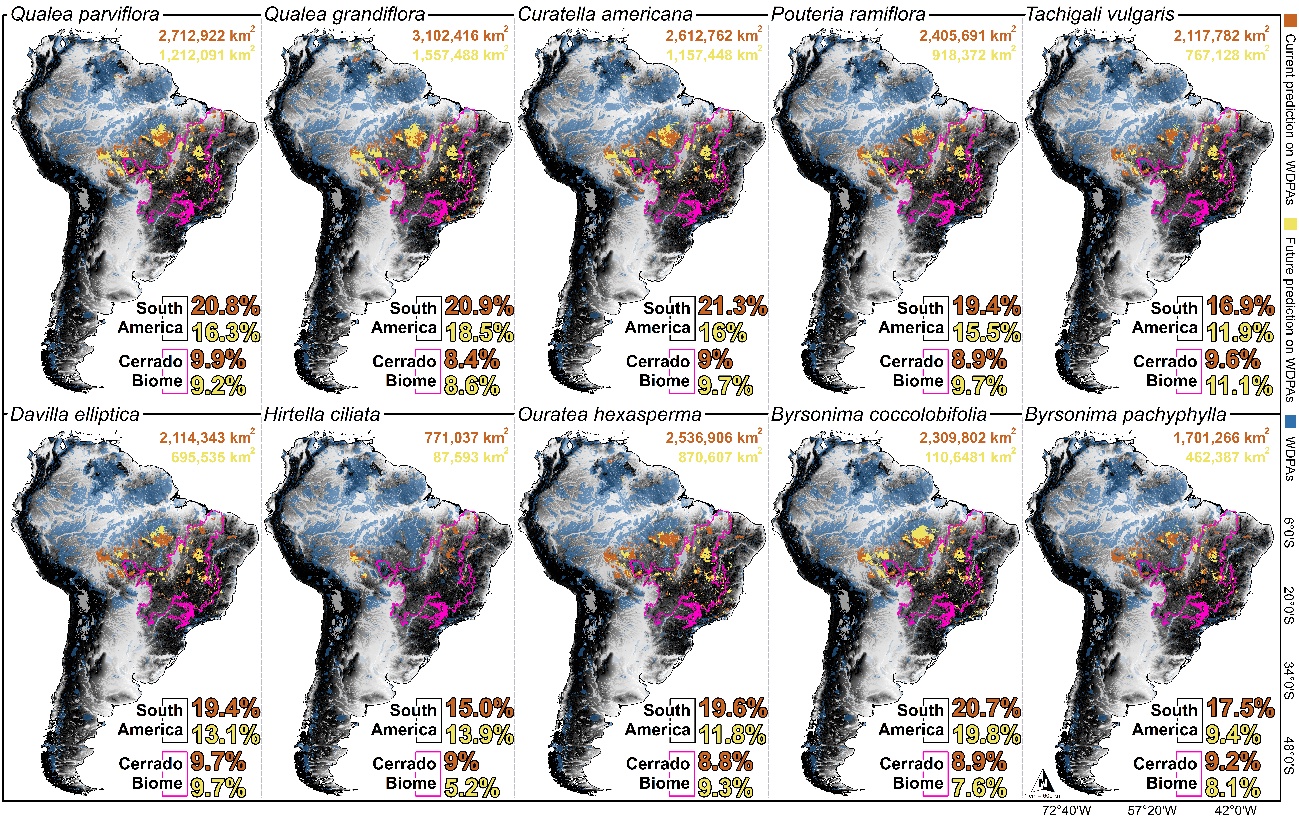


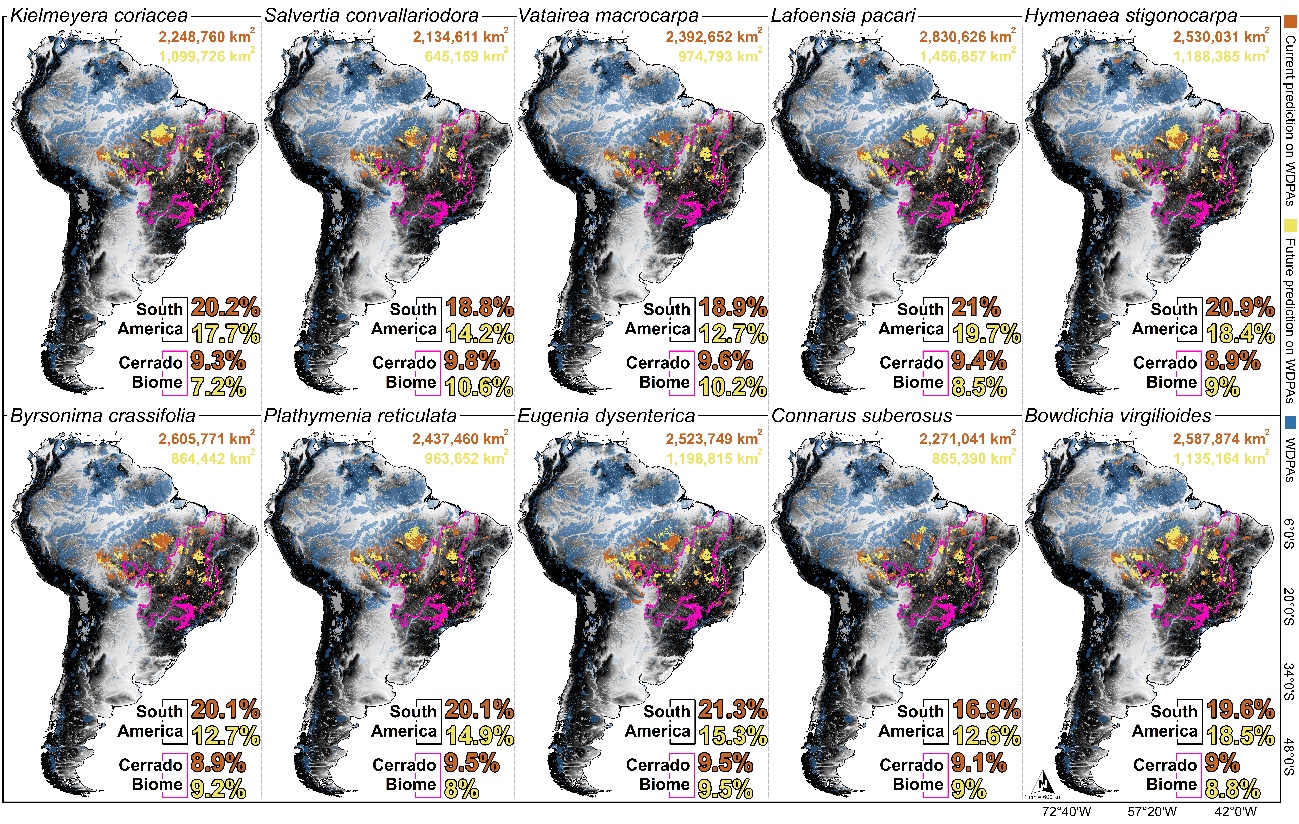


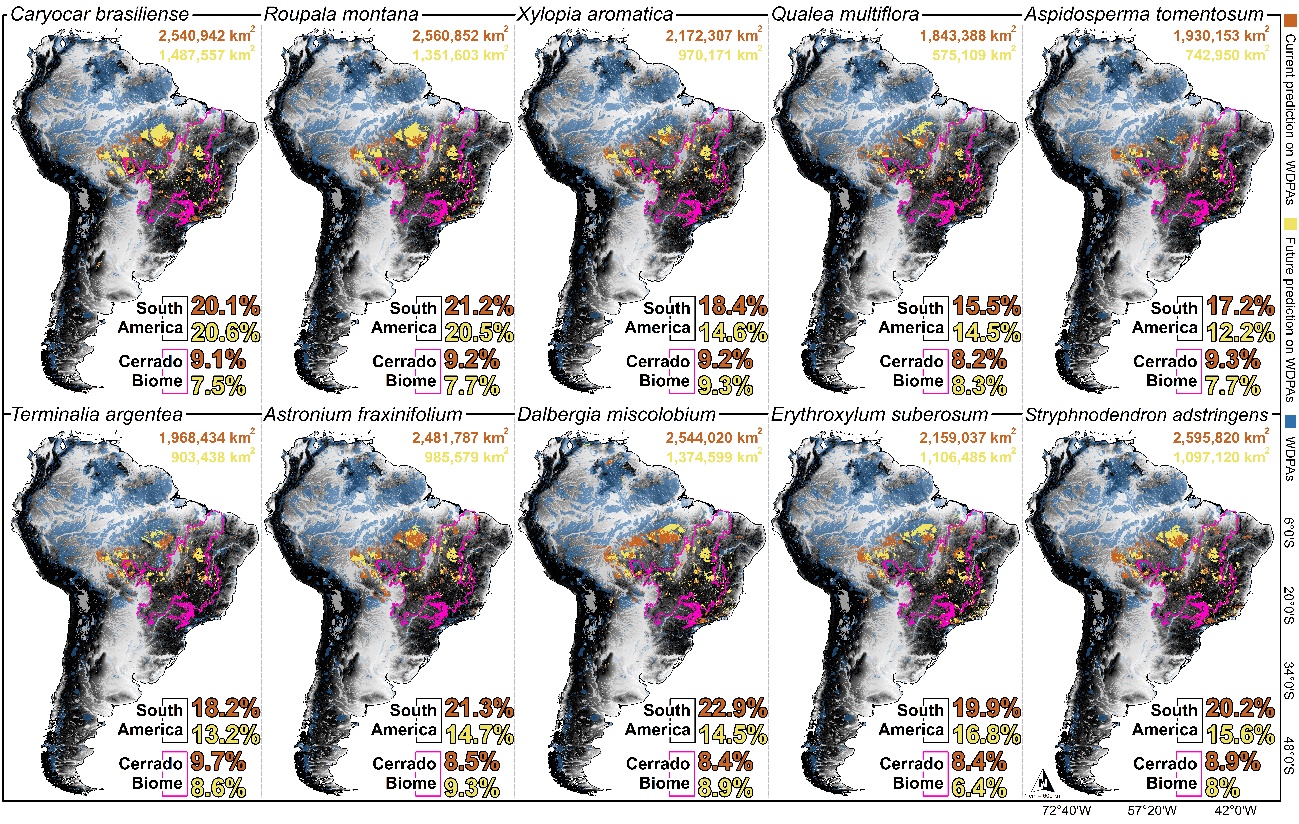


**Supplementary Figure 2:** **Conservation status of the South American savanna ecosystem and the Cerrado biome.** Maps show the geographic overlap between the World Database on Protected Areas (WDPAs, indicated in blue) and the current (orange/brown areas) and future (yellow areas) binary predictions of potential environmental suitability for each Cerrado hyperdominant species. The Cerrado biome is delineated by a magenta outline. Percentage values and areas in $km^2$ indicate the extent of predicted suitability within WDPAs across South America and specifically within the Cerrado biome. Values displayed on the maps are rounded for visual clarity; exact values are reported in Table 1.


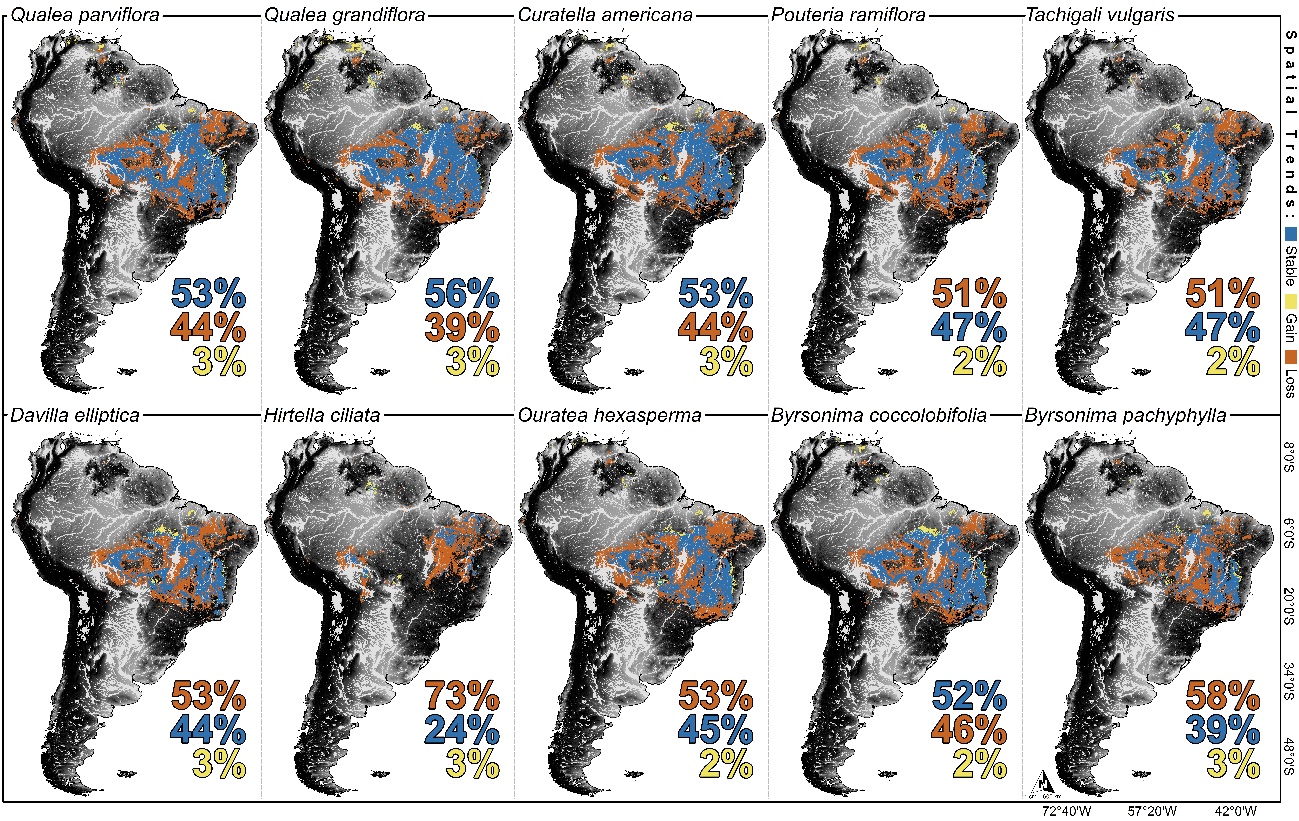


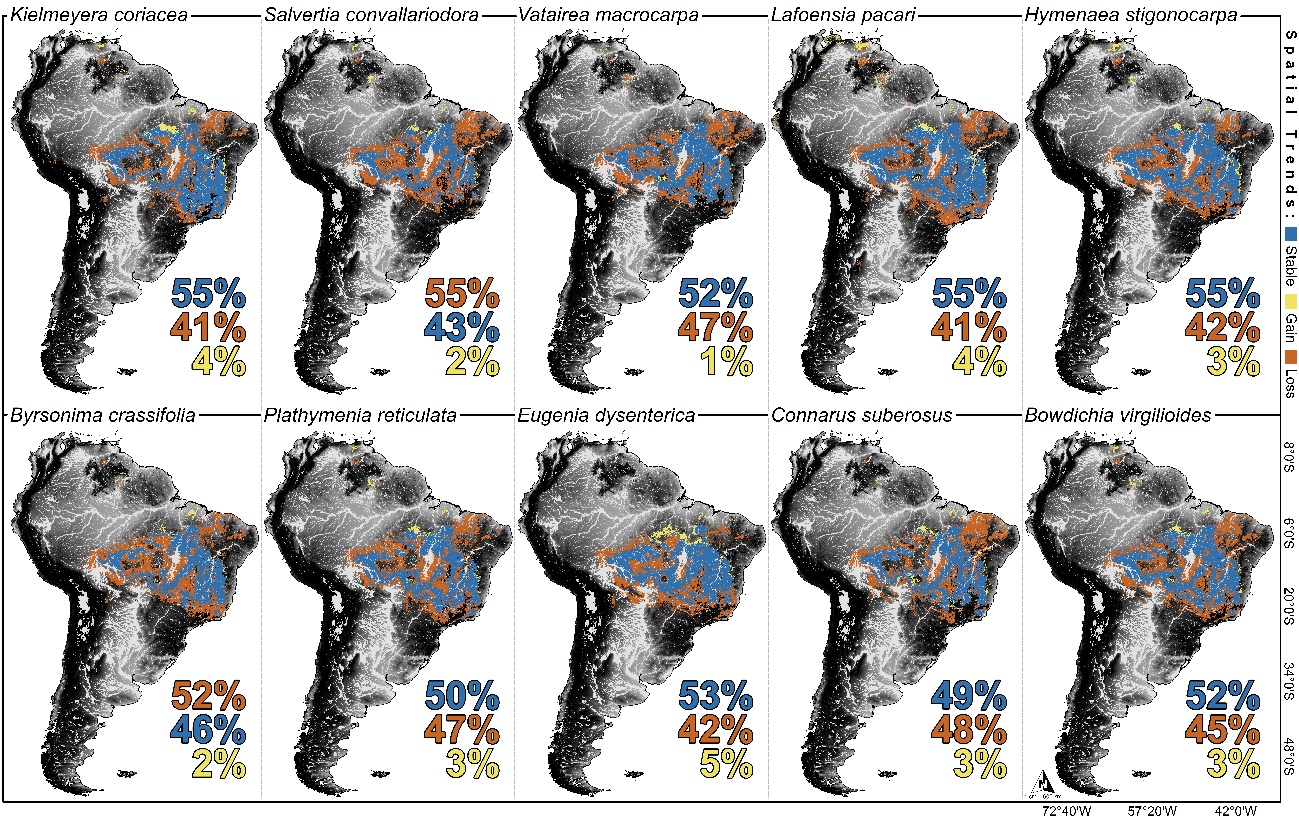


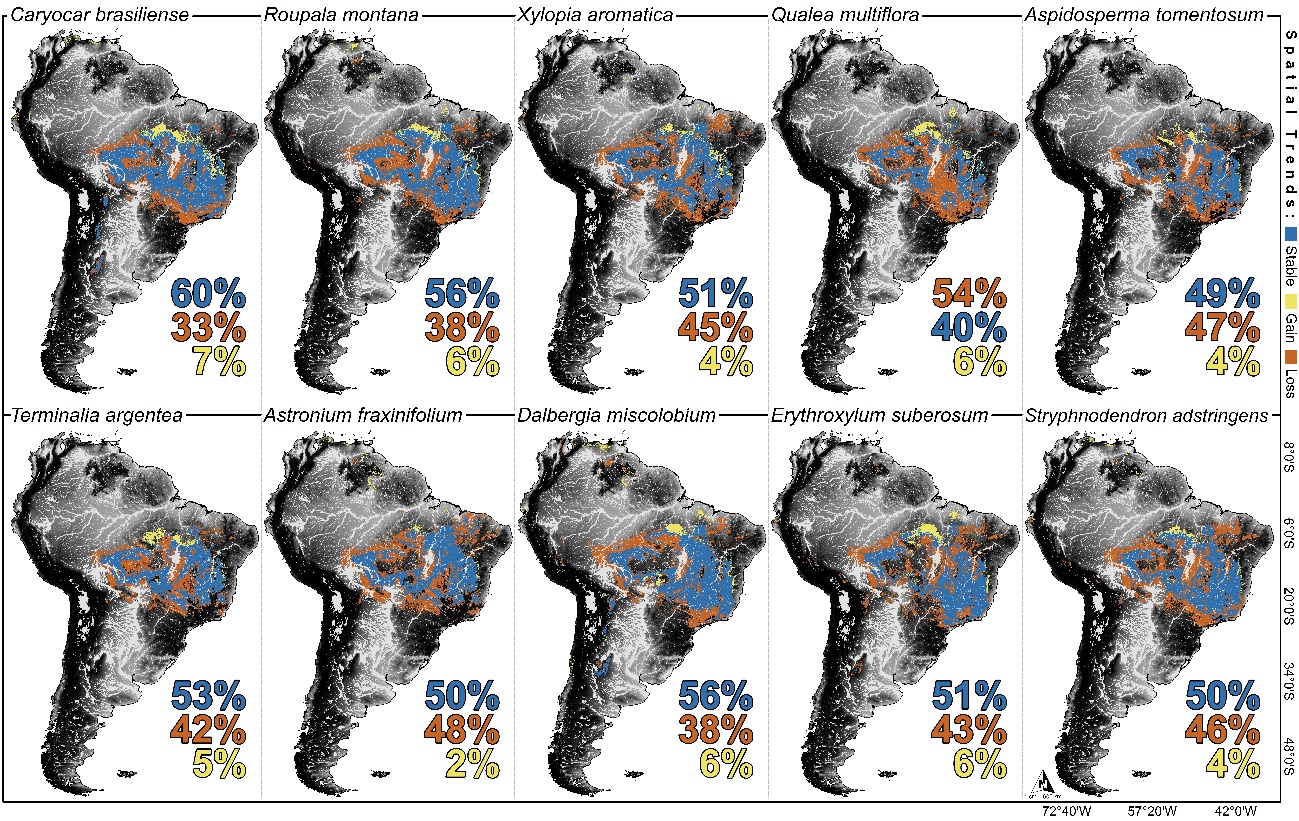


**Supplementary Figure 3: Comparisons between current and future climate scenarios.** Maps show the spatial trends in potential environmental suitability for the 10 Cerrado hyperdominant species across South America. The transitions between current and future scenarios depict areas of stability (blue), loss (orange/brown), and gain (yellow) in potential suitability. Percentage values indicate the proportion of the total projected area corresponding to each spatial trend. Values displayed on the maps are rounded for visual clarity; exact values are reported in Table 1.
