# Supplementary material for: Hyperdominant Trees Reveal Savanna Vulnerability Under Climate Change
Source: Glob Chang Biol. 2026 Apr 16;32(4):e70859. doi: 10.1111/gcb.70859 (PMC13087481; doi:10.1111/gcb.70859)
Supplement: Supplementary file 2 — Table S1: PCA loadings for edaphoclimatic variables and functional traits. [file GCB-32-e70859-s002.zip › Supplementary Information - Table.docx]

**Supplementary Information – Supplementary Table**

**Supplementary Table 1. PCA loadings for edaphoclimatic variables and functional traits.** Current and future variables include precipitation metrics, soil properties, and bedrock depth, while functional traits reflect plant resource-use strategies along acquisitive–conservative axes. Variable loadings indicate the contribution of each factor to the main PCA axes (PC1 and PC2).

|  | **Variables** | **PC1** | **PC2** |
| --- | --- | --- | --- |
| **Current** | Precipitation of Coldest Quarter (bio19) | 0.523 | -0.852 |
|  | Precipitation of Warmest Quarter (bio18) | -0.747 | 0.663 |
|  | Precipitation of Wettest Quarter (bio16) | 0.317 | -0.948 |
|  | Precipitation Seasonality (bio15) | -0.431 | 0.902 |
|  | Depth to bedrock (bio27) | -0.215 | -0.976 |
|  | Absolute depth to bedrock (bio29) | 0.538 | -0.842 |
|  | Silt content (bio50) | 0.186 | 0.982 |
|  | Coarse fragments (bio38) | -0.825 | -0.563 |
| **Future** | Precipitation of Wettest Month (bio13) | 0.052 | -0.998 |
|  | Precipitation Seasonality (bio15) | -0.407 | 0.913 |
|  | Precipitation of Warmest Quarter (bio18) | -0.994 | 0.107 |
|  | Precipitation of Coldest Quarter (bio19) | 0.679 | -0.734 |
|  | Saturated water content (bio20) | 0.920 | 0.391 |
|  | Depth to bedrock (bio27) | -0.148 | -0.988 |
|  | Absolute bedrock depth (bio29) | 0.566 | -0.823 |
|  | Silt content (bio44) | 0.063 | 0.997 |
| **Functional traits** | Leaf fresh mass (LFM) | 0.473 | -0.010 |
|  | Leaf thickness (LT) | 0.427 | -0.060 |
|  | Leaf area (LA) | 0.418 | -0.090 |
|  | Leaf mass per area (LMA) | 0.346 | 0.181 |
|  | Xylem length (XL) | 0.166 | -0.336 |
|  | Outer bark thickness (OBT) | 0.147 | 0.476 |
|  | Fruit width (FW) | 0.141 | 0.473 |
|  | Fruit length (FL) | 0.047 | 0.443 |
|  | Inner bark thickness (IBT) | -0.065 | -0.035 |
|  | Number of seeds (NS) | -0.099 | 0.401 |
|  | Leaf dry matter content (LDMC) | -0.290 | 0.175 |
|  | Bark density (BD) | -0.356 | 0.021 |
