# Supplementary material for: Hyperdominant Trees Reveal Savanna Vulnerability Under Climate Change
Source: Glob Chang Biol. 2026 Apr 16;32(4):e70859. doi: 10.1111/gcb.70859 (PMC13087481; doi:10.1111/gcb.70859)
Supplement: Supplementary file 3 — Appendix S1: Supplementary Methods SM‐1: Modeling and evaluation. SM‐2: Functional traits. [file GCB-32-e70859-s003.docx]

# Supporting Information - Supplementary Methods

# SM-1: Modeling and evaluation

The selected circulation models (CCSM4, MPI-ESM-P, and MIROC-ESM) allow us to represent the range of climate sensitivities and circulation structures among the models available in CMIP5 and include projections for the period 2041–2060, facilitating comparison between different scenarios. For computational reasons and to minimize parsimony in an analysis with 30 species and multiple algorithms, we opted for this small but well-tested set of models and worked with a weighted ensemble of their outputs to express the uncertainty of the predictions. Before the SDM analysis, we resampled all environmental variables (current and future) with a spatial resolution of approximately 5 km at the Equator, suitable for representing edaphoclimatic dynamics and floristic patterns at a macroecological scale (Elith et al. 2006; Barve et al. 2011). We standardized all environmental variables (mean = 0, SD = 1) and limited the spatial extent to South America to avoid bias in species suitability estimates (Barve et al., 2011). To reduce multicollinearity, we conducted principal component analysis (PCA), projected current scenario loadings onto future scenarios, and the first eight components of the PCA explaining 95% of the variance were retained (Supplementary Table 1). The algorithms (MaxEnt, SVM, Domain) were run using block cross-validation and standard settings.

Given the high performance of the SDM family algorithms (Elith et al. 2006), we selected the MaxEnt (ME), Support Vector Machine (SVM), and Domain (DOM) algorithms. Models were implemented in R (R Core Team 2020) using the ENMTML package (Andrade et al. 2020: <https://doi.org/10.6084/m9.figshare.28020971>). We reduced the spatial autocorrelation in occurrence data using the Haversine transformation (distance defined by 2 × cell size). We restricted the accessible area for model calibration using a vector of Neotropical biomes, and delimited the pseudo-absences environmentally, based on the lowest environmental suitability values derived from the Bioclim algorithm.

We trained and validated all models using block cross-validation, running 10 replicates for each algorithm. Following the principle of parsimony, we configured all SDMs using simplified parameters settings to reduce uncertainty associated with model complexity (see Alvarez et al. 2025). We applied ME using linear features, default parameters, 1,000 iterations, 10,000 background points, and logistic output. We fitted SVM using the linear kernel function (standard way) with probabilistic output. We used the *dismo* packages (Hijmans et al. 2017) to run ME, *kernlab* for SVM (Karatzoglou et al. 2004), and *raster* for Domain (Carpenter et al. 1993). We cut the suitability matrices of each algorithm using the Jaccard index. The binary predictions allowed us to identify the areas of presence/absence of the 30 CHT in South America (Barve et al. 2011). Based on the weighted average consensus, we assembled the suitability matrices for the current and future scenarios. We applied a consensus method that considered all models with Jaccard values greater than the average to produce a final distribution for each species.

We evaluated the performance of the models using the True Skill Statistic (TSS) metric, which ranges from -1 and 1, with values ≥ 0.5 or indicating adequate performance. We also evaluated the area under the receiver operating characteristic curve (ROC-AUC) and the Jaccard index. Values from 0 to 1, with values close to 1 showing perfect correspondence between the species' known occurrences and its modeled distribution (Allouche et al. 2006).

**SM-2: Functional traits**

Follow Cruz et al. (2025) the functional traits included: leaf nitrogen concentration (LNC), leaf phosphorus concentration (LPC), leaf area (LA), leaf fresh mass (LFM), leaf dry mass content (LDMC), leaf thickness (LT), leaf dry mass per unit area (LMA), inner bark thickness (IBT), outer bark thickness (OBT), total bark density (TBD), xylem (maximum xylem length: MXL), deciduousness (D), dispersion syndrome [DS: anemocory (A) and zoocory (Z)], fruit [length (L), width (W), consistency (C)] and, N of seeds (NS).

# References

Allouche O, Tsoar A, Kadmon R (2006) Assessing the accuracy of species distribution models: prevalence, kappa and the true skill statistic (TSS). Journal of Applied Ecology, 43, 1223–1232. <https://doi.org/10.1111/j.1365-2664.2006.01214.x>

Alvarez F, Marimon-Junior BH, Marimon BS, Ter Steege H, Phillips OL, Brandão R, Feldpausch TR (2025) Tree species hyperdominance and rarity in the South American Cerrado. Communications Biology, 8(1), 695. <https://doi.org/10.1038/s42003-025-07623-w>

Andrade AFA, Velazco SJE, Júnior PDM (2020) ENMTML: An R package for a straightforward construction of complex ecological niche models. Environmental Modelling & Software, 125, 104615. <https://doi.org/10.1016/j.envsoft.2019.104615>

Barve N, Barve V, Jiménez-Valverde A, Lira-Noriega A, Maher SP, Peterson AT, Villalobos F (2011) The crucial role of the accessible area in ecological niche modeling and species distribution modeling. Ecological Modelling, 222(11), 1810-1819. <https://doi.org/10.1016/j.ecolmodel.2011.02.011>

Carpenter G, Gillison AN, Winter J (1993) DOMAIN: a flexible modelling procedure for mapping potential distributions of plants and animals. Biodiversity and Conservation, 2(6), 667–680. <https://doi.org/10.1007/BF00051966>

Cruz WJA, Marimon BS, Marimon-Junior BHM, Morandi PS, Longhi SG, Prestes NCCDS, Ribeiro-Júnior NG, Reis SM, Scalon MC, de Oliveira EA, Levesley A, Phillips OL (2025) Functional Biogeography and Ecological Strategies of Trees Across the Amazon–Cerrado Transition. Journal of Vegetation Science, 36(5), e70076. <https://doi.org/10.1111/jvs.70076>

Elith JH, Graham CP, Anderson R, Dudík M, Ferrier S, Guisan A, Zimmermann, N (2006) Novel methods improve prediction of species’ distributions from occurrence data. Ecography, 29(2), 129-151. <https://doi.org/10.1111/j.2006.0906-7590.04596.x>

Hijmans RJ, Phillips S, Leathwick J, Elith J (2017) dismo: Species Distribution Modeling. R package version 1.1-4. [https://CRAN.R-project.org/package=dismo](https://cran.r-project.org/package=dismo)

Karatzoglou A, Hornik K, Smola A, Zeileis A (2004) Kern-lab - An S4 package for kernel methods in R. Journal of Statistical Software, 11:1–20. <https://doi.org/10.18637/jss.v011.i09>

R Core Team (2020) R: A language and environment for statistical computing. R Foundation for Statistical Computing, Vienna, Austria. ISBN 3-900051-07- 0. [http://R-project.org](http://www.r-project.org)

Schwalm CR, Glendon S, Duffy PB (2020) RCP8.5 tracks cumulative CO2 emissions. Proceedings of the National Academy of Sciences, 117(33), 19656-19657. <https://doi.org/10.1073/pnas.2007117117>
